# Multidisciplinary oil spill modeling to protect coastal communities and the environment of the Eastern Mediterranean Sea

**DOI:** 10.1038/srep36882

**Published:** 2016-11-10

**Authors:** Tiago M. Alves, Eleni Kokinou, George Zodiatis, Hari Radhakrishnan, Costas Panagiotakis, Robin Lardner

**Affiliations:** 1 3D Seismic Lab–School of Earth and Ocean Sciences, Cardiff University–Main Building, Park Place, Cardiff, CF10 3AT, United Kingdom; 2 Department of Environmental and Natural Resources Engineering, Technological Educational Institute Crete, 3 Romanou Str. Chalepa, Chania, Crete GR 73133, Greece; 3 Oceanography Centre, University of Cyprus, P.O. Box 20537, 1678 Nicosia, Cyprus; 4 Department of Business Administration, Technological Educational Institute Crete, Agios Nikolaos, Greece

## Abstract

We present new mathematical and geological models to assist civil protection authorities in the mitigation of potential oil spill accidents in the Eastern Mediterranean Sea. Oil spill simulations for 19 existing offshore wells were carried out based on novel and high resolution bathymetric, meteorological, oceanographic, and geomorphological data. The simulations show a trend for east and northeast movement of oil spills into the Levantine Basin, affecting the coastal areas of Israel, Lebanon and Syria. Oil slicks will reach the coast in 1 to 20 days, driven by the action of the winds, currents and waves. By applying a qualitative analysis, seabed morphology is for the first time related to the direction of the oil slick expansion, as it is able to alter the movement of sea currents. Specifically, the direction of the major axis of the oil spills, in most of the cases examined, is oriented according to the prevailing azimuth of bathymetric features. This work suggests that oil spills in the Eastern Mediterranean Sea should be mitigated in the very few hours after their onset, and before wind and currents disperse them. We explain that protocols should be prioritized between neighboring countries to mitigate any oil spills.

Marine pollution in the European Seas resulting from maritime traffic or offshore drilling is regulated by multiple institutions and conventions^1^, in which are included: (a) the MARPOL 73/78 international convention; (b) the OSPAR Convention of 1992 for the North-East Atlantic, (c) the Baltic Convention of 1992, and (d) the Barcelona Protocol of 1994 for the Mediterranean Sea. Against such a backdrop, the Mediterranean region has witnessed a recent phase of industrialization due to an ever-increasing need for energy and mineral resources from European and American nations, and anticipating a sharp demographic growth in SE Asia and Africa^2^. Oil and gas (condensate) fields have been explored for, and discovered, in the past five years offshore Cyprus, Egypt and Israel^3^. In parallel, the Suez Canal has expanded its capacity being now able to expedite oil tankers up to 550,000 deadweight (dwt) in volume. Nearby refineries and ports have been upgraded to deal with the expected increase in ship tonnage and volumes of oil and gas produced by new offshore fields^4^. Yet, East Mediterranean countries have found themselves without efficient mitigation plans, and with no combined real-time surveying technology to help civil protection authorities in oil spill containment and cleansing^5^.

Water circulation in the Eastern Mediterranean Sea has been shown to be complex and energetic with several alternating cyclonic and anticyclonic eddies, gyres and jets^6^,^7^. In the regions where the examined offshore and coastal platforms are located, the main flow features are those of the meandering cross-basin jet, known as the Mid Mediterranean Jet-MMJ, the Cyprus anticyclonic eddy, the anticlockwise current along the coastal zone of Egypt, Israel and Lebanon and other currents smaller in scale, but with highly variable eddies^8^,^9^,^10^. Knowing the position and strength of these circulation patterns is critical to oil spill predictions, as the apparently permanent flow features of the region change in time, space, shape, size and intensity on a temporal scale of weeks to months^9^,^10^,^11^. As shown in ref. 12, the combined action of NW winds turning SW, and of local sea currents in respect to the location of the Cyprus eddy activity, resulted in the capture of an oil spill hypothetical released from the Aphrodite well, within the area of the Cyprus eddy, for a period of more than a month, not allowing the movement oil slick to the coast of nearby countries. Notwithstanding these findings, the Cyprus eddy shows a striking variability in terms of its location, within a distance of around 80 km and a period of 2–3 months.

The present study shows for the first time the long-term daily and bi-weekly variability of oil spill predictions for the Eastern Mediterranean Sea, using meteorological and oceanographic data of high spatial and temporal resolution. This study demonstrates the importance of major flow features dominating the Eastern Mediterranean Sea in the dispersion of oil spills from each existing offshore and coastal platforms (Fig. 1). The nineteen (19) oil spill locations selected for modeling are, in the whole of the Eastern Mediterranean Sea, where the heaviest shipping traffic (and related oil spill incidents) are more frequently recorded, and where hydrocarbon exploration and production are more intensive (Fig. 1 and Supplementary.kzm file). As the Eastern Mediterranean constitutes an environmentally protected area of the highest priority^13^, it was crucial to prepare our modeling work flow by: (a) taking into account the high temporal and spatial variability in oceanographic and meteorological conditions recorded in the Eastern Mediterranean Sea, namely with the use of a high-resolution database spanning from 2009 to 2014, (b) gathering detailed bathymetric and geomorphologic data to assist the compilation of hazard maps^5^ for the Eastern Mediterranean Sea, knowing in advance that large swathes of the coastline are low-lying, sediment rich and thus vulnerable to the smallest of pollution events, (c) gathering new geomorphologic and geologic data from the ArcGIS 10 online database, Google Maps Pro^®^ and repositories in Greece and Cyprus. The latter data will allow to risk (or de-risk) parts of the coastline, and thus concentrate civil protection teams in areas where mitigation techniques are not only necessary, but urgent. A key driver for this work was the need to provide a benchmark methodology to assess oil spills in confined maritime basins, which record ever-greater ship traffic and industrial pressures in several parts of the world, including in the Eastern Mediterranean Sea where more than 73 oil spills have been recorded from 1977 to 2013 (Fig. 1 and Supplementary Table 1). In addition, modeling of pollutant dispersal is equally important before and after the application of mitigation techniques^14^,^15^.

## Methods

The methodology used in this work was developed under the umbrella of European Commission projects concerning oil spill response and predictions (www.nereids.eu, www.myocean.eu, www.medess4ms.eu, www.raop.eu; www.emodnet-mediterranean.eu). Bathymetric, geomorphological, meteorological and oceanographic parameters were considered, in a first stage, as the main factors controlling the dispersion of oil slicks in the Eastern Mediterranean Sea^5^,^12^,^16^–19. Oil dispersion was simulated in a second stage using MEDSLIK^20^ and high temporal and spatial resolution data for wind, wave, sea surface temperature, together with 3D sea-current conditions provided by the CYCOFOS downscaled from the Copernicus-Marine environment monitoring service (CMEMS) marine service. The spatial and temporal resolution of the meteorological-oceanographic data used in the present MEDSLIK oil spill simulations are 3 hourly, 1.8 km resolution for sea currents; and 3 hourly, 5 km resolution for winds and waves. Oil fate models were also generated to estimate the percentage volumes of oil evaporated, dispersed and trapped on Eastern Mediterranean shores. A volume of 55,800 bbls of medium-grade Belayim Oil was selected for the models based on a request from the Regional Marine Pollution Emergency Response Centre for the Mediterranean Sea (REMPEC), following the MEDEXPOL 2013 workshop^21^. The Belayim blend is produced in the Gulf of Suez and comprises a medium crude oil with a specific gravity of 26° API. Specifically, the basic steps of the methodology applied were:

Step 1. Bathymetric features are known to be able to alter ocean circulation^17^,^18^,^19^. For this reason, we calculated^22^,^23^ the slopes (Fig. 2a) and aspects (Fig. 2b) of the bathymetric data provided by EMODNET^24^. In our calculations of slopes, *S*(*p*) = tan^−1^(|*v*(*p*)|), corresponds to the slope at point p of the topographic surface Z, whereas *v*(*p*) denotes the plane tangent vector defined as 
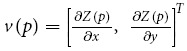
. Slope is measured in degrees as (*p*) ∈ [0, 90°]. In addition, 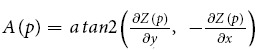 corresponds to the aspect at point p of the topographic surface Z. Aspect is measured in degrees as *A*(*p*) ∈ [0, 360°]. The above calculations have been undertaken using Matlab. The calculated slopes and aspects have been exported in.shp (shape) format in order to further been processed in ArcGIS 10.

Step 2. Taking into account the results obtained in Step 1 (see Supplementary Table 2, columns 4, 5, 6) and the geographic dispersion of the spills (Fig. 3 and Supplementary Figures 1 to 19) we defined seven (7) sub-regions with different morphologic characteristics (Fig. 2).

Step 3. In this third step, we selected the maximum trajectory (maximum area covered) of the spills (Fig. 3 and Supplementary Figures 1 to 19) and created a database in ArcGIS 10 that included the bathymetry, the slopes, the aspects and the spill trajectories.

**Figure 1:**
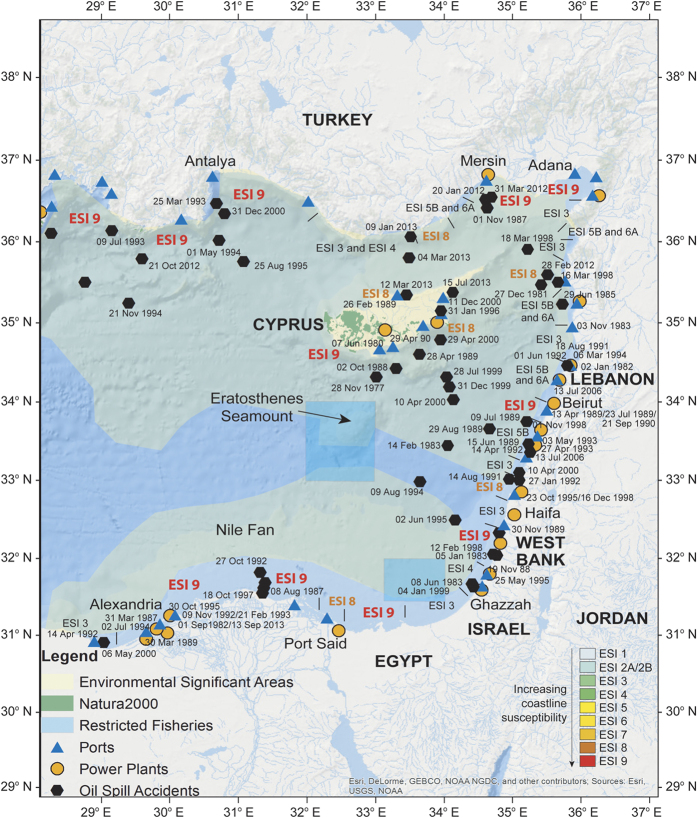
General map of the study area showing the location of environmentally significant areas, Natura 2000 sites in Cyprus, areas of restricted fishing, ports, power plants and recorded oil spill incidents, East of 29^o^E, from 1977 to 2013. Coastline susceptibility values for the Eastern Mediterranean are shown together with the location and dates of historic oil spills. The figure has been created using the ArcGIS 10 (http://www.esri.com/news/arcnews/spring12articles/introducing-arcgis-101.html). The base map is from the free access ArcGIS 10 online database (https://doc.arcgis.com/en/arcgis-online/share-maps/supported-items.htm). Natura 2000 data are from http://www.eea.europa.eu/data-and-maps/data/natura-2/natura-2000-spatial-data/natura-2000-shapefile-1. Locations of ports and historical oil spill accidents are from the MEDESS 4MS access system at http://www.medess4ms.eu/ and (http://medgismar.rempec.org/#) as shown in Supplementary Table 1.

Step 4. Finally, we undertook a qualitative assessment of the relation between oil spill trajectories and seabed morphology (see Supplementary Table 2) and evaluated shoreline susceptibility (Fig. 1 and Supplementary Table 1) around the Levantine Basin^5^,^25^.

**Figure 2:**
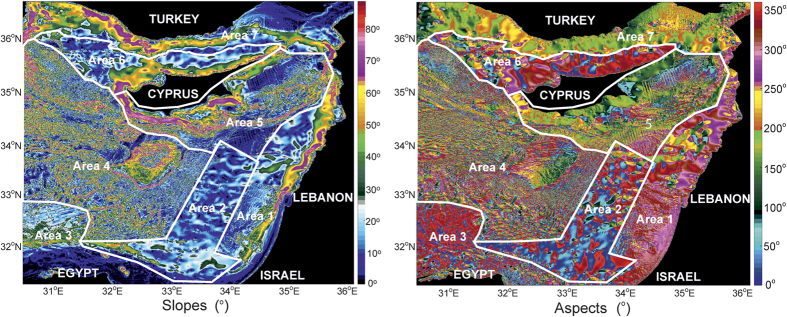
Bathymetric data for the Eastern Mediterranean showing: (**a**) slope gradients and (**b**) aspects. The study area was divided in seven (7) distinct zones based on recorded slope gradients and aspects. Note the predominant Erastosthenes Seamount to the south of Cyprus. The figure was created on Matlab-r2010a (http://matlab-r2010a.software.informer.com/). Bathymetric data are from the open database EMODnet (http://www.emodnet-hydrography.eu/).

**Figure 3:**
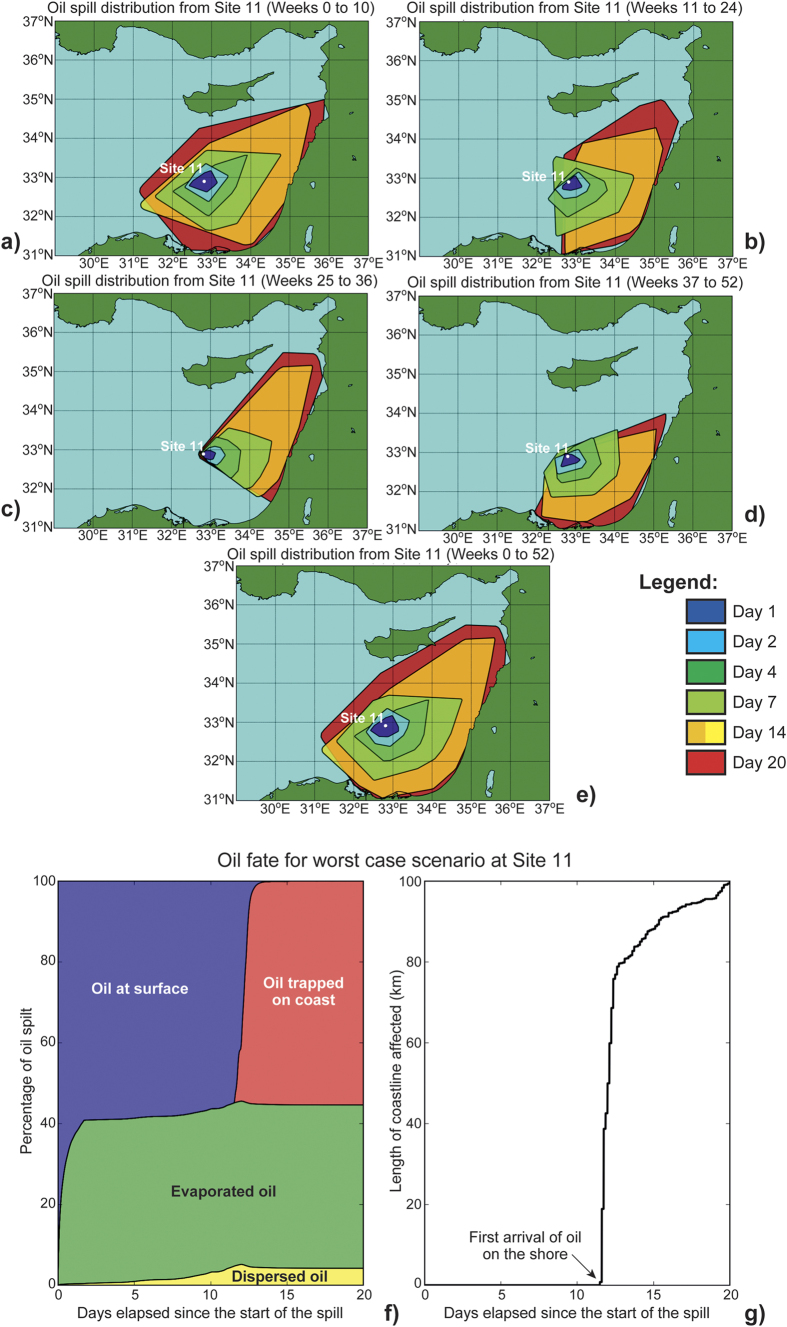
Example of MEDSLIK oil spill simulations undertaken for Site 11, located at the Zohr Field, close to the boundary between Cyprus and Egypt waters (see Fig. 4 for location). The figure shows the modeled distribution of oil slicks (in days) for: (**a**) weeks 0–10; (**b**) weeks 11–24; (**c**) weeks 25–36; (**d**) weeks 37 to 52; (**e**) weeks 0–52. (**f**) Highlights the percentage of oil dispersed, evaporated, trapped on coast and at surface for data from weeks 0–50. (**g**) Shows the length of affected coastline for the scenario in (**e,f**). The figure was compiled using MEDSLIK (http://www.oceanography.ucy.ac.cy/research/oil-spill-modeling/medslik/).

**Figure 4:**
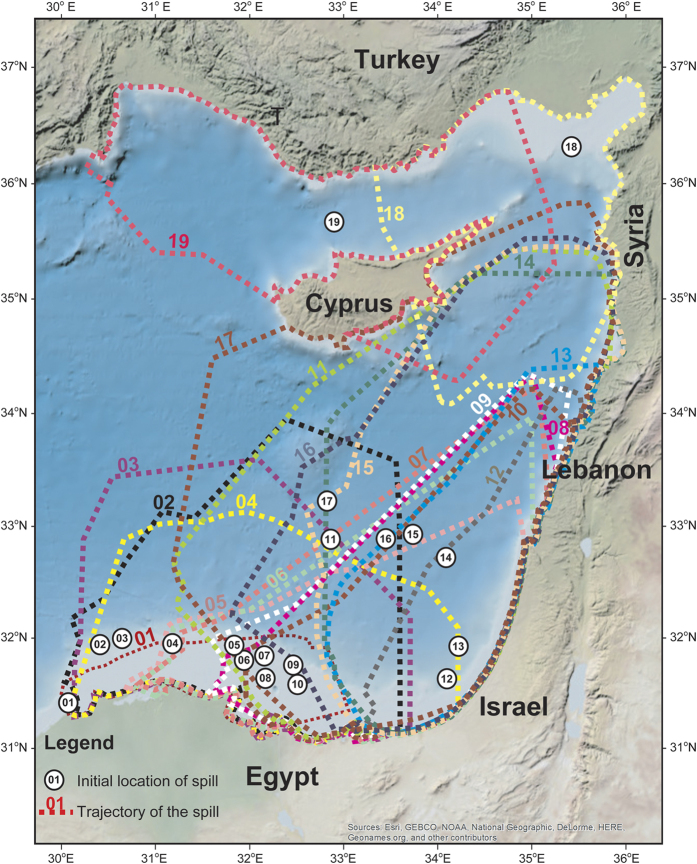
Overlay map showing the locations of oil release (white circles with numbers) of the 19 modeled oil spills, and their dominant trajectories. The map was compiled on ArcGIS 10 (http://www.esri.com/news/arcnews/spring12articles/introducing-arcgis-101.html). Topographic and bathymetric data for this basemap are from the free access ArcGIS 10 online database (https://doc.arcgis.com/en/arcgis-online/share-maps/supported-items.htm). Trajectories of oil spills have been digitized using the results of the MEDSLIK models produced for this work (Fig. 3 and Supplementary Figures 1 to 19).

The adopted methodology is summarized in Figs 2, 3 and 4 and as supplementary data (Supplementary Table 2 and Supplementary Figures 1 to 19). As an example of our methodology, we consider the oil leak in Site 01, offshore north of Egypt, shown in Fig. 4 (white circle indicated with number 1). In this area, the prevailing bathymetric slopes (Fig. 2a) are 0°–10° (i.e., an almost flat seabed), while the bathymetric features generally dip to the North (prevailing aspects 0°–25° (N-NNE) and 345°–359° (NNW-N) in area 3, Fig. 2b). In addition the prevailing direction, relative to the North, of the bathymetric features in this area is E-W (almost vertical to the aspect), and is in agreement with the major axis of the expanded oil spill (see the trajectory of the oil spill for Site 01 in Supplementary Figure 1). Finally, according to the oil spill simulation for Site 01 (Supplementary Figure 1), in the first two (2) days after the spill the oil slick is expected to remain at surface and evaporate. Oil spill dispersion presents very low values, at ~1% percent. The oil slick remains at the surface until the 4th day, while continuous evaporation and expansion to the coast takes part in the next few days. The susceptibility of the shoreline in this area is very high (ESI 9, Fig. 1).

## Results

### Morphological analyses

The results of previous studies^17^,^18^,^19^,^26^ strongly support that seabed morphology is able to alter the hydrodynamic regime changing the water circulation pathways and affecting the sedimentary processes. Slopes in the Eastern Mediterranean Sea are generally steep, exceeding 40°, but with gentle continental shelves, seamount tops and abyssal areas on which slope gradients vary between 0° and 20° (Fig. 2a and Supplementary Table 2, column 4). The northeastern part of the study area, offshore Turkey and Syria comprises one of these regions showing gentle slopes (Fig. 2a). Slope morphology changes again towards Israel and Lebanon, where a steep continental slope is observed (Fig. 2a). Prevailing aspects in the study area are 0°–25° (N to NNE), 125°–175° (SE to S), 180°–250° (S to SW), 275°–300° (W to NW) and 345°–359^o^ (NNW to N) (Fig. 2b and Supplementary Table 2, column 5). Furthermore, the general orientations of seabed features in the central and southern part of the Eastern Mediterranean Sea are E-W and NE-SW, whereas the northern part shows E-W and ENE-WSW structural trends on the seafloor (Fig. 2b and Supplementary Table 2, column 6). The oil spill trajectories mainly present NE-SW (14 cases) and E-W (4 cases) orientations (with respect to the North) for their major axis (Supplementary Table 2, column 3). In all cases examined, the major axis of spill’s trajectories is consistent with the prevailing orientation of the seabed morphology in the area where the oil spill is expanded (Supplementary Table 2, columns 3 and 6).

### MEDSLIK modeling results

A series of nineteen (19) oil spill scenarios shown in Supplementary Figures 1 to 19 (an example is shown in Fig. 3 for the recently discovered Zohr Gas Field), have been prepared for week to week conditions for a period of four (4) years, presenting the detailed trajectories of the spills together with graphs corresponding to the percentage volumes of dispersed, evaporated, trapped and at the surface, one (1) to twenty (20) days after the onset of the spill (Fig. 4 and Supplementary Table 2). As previously mentioned, the modeled spills took into account the release of 55,800 bbls of medium-grade Belayim Oil, the common type produced in the region, following the REMPEC MEDEXPOL 2013 experiment^21^. Figure 4 and Supplementary Table 2 show that the major axes of oil spills will be oriented NE-SW or E-W, while minor axes will trend SW-NE or N-S.

Currents and eddies in the Eastern Mediterranean Sea are controlled by a strong thermohaline pattern, in contrast to Ekman currents in regions such as the North Atlantic and other open seas^9^,^27^. The upper surface layer is controlled by the prevailing wind patterns, which are essentially northwesterly and southwesterly in the Eastern Mediterranean^7^,^28^. Oil spill trajectories between Cyprus and Egypt are markedly influenced by the Eratosthenes Seamount, around which the circulation pattern form a regional eddy from the sea surface down to 400–500 m, known as the Cyprus warm core eddy. As result, the MEDSLIK models show the most vulnerable areas to be the eastern part of the Levantine Basin, offshore Israel and Lebanon, as well as the Cyprus shorelines (Fig. 4). These regions will be affected approximately four (4) to fourteen (14) days after oil spill accidents in Egyptian waters (Fig. 4 and Supplementary Table 2). In such a setting, the coasts of eastern Egypt and Israel are the most likely to be affected by a spill in 18 out of the 19 scenarios modeled in this work (Fig. 3 and Supplementary Figures 1 to 19). In contrast, not all modeled spills are mobile enough to reach Turkey and Syria, eventually affecting beaches in Israel and Lebanon before dissipating and evaporating completely (Fig. 4). In fact, spills that occur close to the Egyptian coastline, or in shallow proximal parts of the Nile Delta (Fig. 4, Sites 01–11), will disperse and affect the Egyptian coast, from west to east, without reaching Israel (Figs 1 and 4). Concerning the fate of oil in the study area, the modeled oil slicks remain at the surface and are evaporated within a time period of 2–6 days, often prior to reaching the coast (Supplementary Table 2, column 8). However, the length of the coast affected by the remaining oil is considerable large; ~20–85 km in most cases and up to 116 km in the most extreme scenario (Supplementary Table 2, column 7). The extend of the MEDSLIK modeling results regarding the coastline affected by oil spills was documented to coincide with *in-situ* observations during the so far biggest oil pollution crisis in the Eastern Mediterranean, that of the Lebanese oil pollution of summer 2006^15^.

### Shoreline susceptibility analysis

In the Eastern Mediterranean, the nature of shoreline sediments is related to the shoreline morphology. The geomorphological mapping undertaken in this work reveals a straight coastline between the Al-Manzalah Lake (Egypt) and central Israel (Fig. 1). A linear coastline is also observed from Lake Bardawil to Haifa (Fig. 1). However, the shores of Israel and Lebanon are exposed to wave action, a phenomenon promoting the natural cleansing of oil affecting beaches and harbours^5^,^25^. Spits and bays, usually forming local traps for spilt oil, occur from Haifa towards Lebanon, where rocky shorelines are common. A similar shoreline morphology is maintained in Syria and Turkey despite the presence of linear, low-lying beaches south of Tartus (Syria), Latakia (Syria) and in southern Turkey (Fig. 1). Using the ESI coastal susceptibility classification^25^, we notice a marked change in susceptibility at the latitude of Haifa (Fig. 1). To the south of Haifa, susceptibility values are relatively low (ESI 3 to ESI 4), bar the Ashkelon National Park (Israel) and the areas with coastal lagoons in Egypt (Fig. 1). To the north of Haifa, and in Cyprus, large swathes of the shoreline reach values of ESI 8 and ESI 9 (Fig. 1).

## Discussion

### Environmental management of the Eastern Mediterranean as a future energy hub

The present work shows that the Eastern Mediterranean Sea presents high risk in terms of oil pollution, rendering the use of oil spill models crucial for the area. There are several reasons for the increased risk observed in the study area. First, the average age of vessels calling at Limassol, Alexandria, Valletta and Mersin is over 20 years compared to less than 14 years at the West Mediterranean ports of Algeciras, Augusta, Palma, Barcelona, Genoa, Fos and Gibraltar^29^. In view of the correlation between vessel age and casualty risk, the deployment of older tankers in the Eastern Mediterranean potentially exposes this area to a greater risk of a casualty-related pollution event^30^.

Recent studies estimate a worldwide marine traffic increase of 10% up to 2018, with an estimated increase of 23% in the Mediterranean Sea alone^30^. Such a marine traffic will lead to increasing risks in well-known traffic choke-points such as the Suez Canal in the Eastern Mediterranean, the Sicilian Strait in the Central Mediterranean and the Gibraltar Strait in the Western Mediterranean. Other regional developments include: a) the construction of the Vasilikos oil terminal in Cyprus, the first terminal of its kind in the Eastern Mediterranean - the terminal currently comprises 28 tanks and capacity of 544,000 m^3^ with plans to expand to host vessels up to 850,000 m^3^ in net volume; and b) the finding of the Zohr field in Egypt’s waters, adding to the already developing oil and gas production offshore Israel and the discovery of new hydrocarbon fields in Cypriot, Egyptian and Israel waters (Figs 1, 3 and 4). This will result in a significant increase in oil and gas operations and the construction of new oil and gas pipelines in the Eastern Mediterranean, rendering the need for oil spill model use more crucial to oil spill monitoring and mitigation. With that in mind, the models presented in this work can be used in the guidance of mitigation teams, and in the establishment of protocols between neighboring countries.

### Oil contingency measures

Oil transport activity through the Suez Canal and the high environmental and social capital of the Southeast Mediterranean Sea renders the area a high risk for oil pollution. In the last decade, only seven (7) serious marine accidents have been recorded (Supplementary Table 1) but more than 1000 possible oil spills were detected in the Levantine Basin through satellite observation systems^31^. The potential increase in shipping traffic and the recent discoveries of large gas fields in the offshore region between Israel, Egypt and Cyprus will increase oil spill risk, therefore urging the implementation of both national and international Contingency Plans for combating oil spills.

Oil spill contingency planning must start with the clear definition of assets and operations to be included within the planning scope^31^,^32^. This signifies, in practice, that industries such as shipping, pipelines, ports, oil handling facilities, and exploration and production operators differ widely in their scale of operational activities, environmental concerns, regulatory requirements and consequent oil spill risks. With that in mind, the Cyprus government has developed a three tiered structure allowing those involved in contingency planning to understand how effective a response to any oil spill will be; from small operational spillages to a worst-case release at sea or on land. The structure also provides a mechanism to identify how individual elements of capability will be cascaded down to the field of operations, i.e. where the oil spill effectively is.

Tier 1 capabilities describe the operator’s locally held resources used to mitigate spills that are on or near an operator’s own facility. The resources also provide an initial response to spills that may potentially escalate beyond the scope of Tier 1 initial actions and capabilities.

Tier 2 capabilities refer to additional, shared, national or regional resources necessary to supplement a Tier 1 response or support an escalating response. Tier 2 capability includes a wider selection of equipment and expertise suited to a range of strategic response options.

Tier 3 capabilities are globally available resources that further supplement Tiers 1 and 2. They comprise the international resources necessary for spills that require a substantial external response due to incident scale, complexity and/or impact potential.

After a range of oil spill planning scenarios are selected, consideration shifts to the development of appropriate response strategies, i.e., to available and viable response techniques that adequately mitigate the impact and consequences of each scenario. Planners should therefore consider how the response to a scenario might develop over time and how the strategy may need to adjust as the spill evolves. The strategy to be adopted should be focused on clear, attainable goals by first establishing a set of response objectives for the planning scenarios. Objectives are based on a number of inputs. However, those objectives that are largely common to all spill scenarios are to: (a) protect the health and safety of responders and the public; (b) control the source; (c) contain and recover spilled material; (d) maximize the protection of sensitive areas; and (e) minimize damage to environmental and socio-economic resources. Ηealth, safety and, in certain circumstances, security considerations are a significant part of a response and are always the top priority.

### Oil combating measures and net environmental benefit analyses

The identification of sensitive resources and priority protection sites, as determined by the sensitivity mapping, provides the site-specific information to inform net environmental benefit analysis (NEBA) discussions and develop strategies that best meet the objectives of sensitive area protection and the minimization of damage. This signifies that scenarios that are more complex may require one or multiple strategies consisting of various combinations of techniques at different tier levels, possibly in different locations or for varying seasonality. For example, a scenario with the potential to occur both in winter and in summer when open water conditions exist will likely require multiple strategies, as the preferred mitigation techniques will change due to the seasonal conditions. A scenario with the potential for impact on offshore, near-shore and shoreline areas will require a strategy with a variety of techniques suitable for use in those unique environments. At present, the types of response resources that should be described include, but are not limited to:

- Spill response equipment (booms, skimmers, barges, skimming vessels, etc.);
- Logistical support service providers and equipment/supplies;
- Vessels of opportunity (required vessel specifications, lists of locally available vessels, etc.);
- Local labour sources and volunteers; and
- Subject matter experts.

Once the most effective and feasible response techniques are identified for each scenario, a NEBA must be carried out to determine which of those technique(s) will have the greatest net environmental benefit. The NEBA process provides a means for selecting the best response actions that minimize potential impacts on people and the environment. It presents a useful framework to achieve science-based planning and stakeholder consensus prior to, and away from, the emotive atmosphere prevalent at the time of a spill. The NEBA uses the planning scenario information-including data on the environmental and socio-economic resources identified in the sensitivity mapping process-experience from previous spills, and scientific expertise to inform an assessment of the environmental and social impacts that could potentially result from the use of certain response techniques at specific locations. The NEBA process weighs the advantages and disadvantages, or trade-offs, of the available techniques so that an effective response may be formulated to achieve the maximum overall benefit for the environment. Finding consensus is an important part of the process; conflicts do occur, and an informed discussion should take account of the various stakeholder priorities and concerns that may be raised at each location.

Natural recovery (i.e. no human intervention) is used as the benchmark against which to evaluate response actions. For example, if the use of intensive clean-up techniques on remote shorelines is not going to bring meaningful socio-economic benefits, or if it has the potential to exacerbate the ecological damage, its validity should be questioned. Such considerations should take account of the recreational, economic and wildlife uses of the shoreline, the safety of the public and responders, and the possibility of bulk oil remobilizing and spreading the contamination further afield.

Through the use of NEBA, the relevant stakeholders in contingency planning should be able to understand the reasons why certain response options are included in the response strategy. If regulatory approval for a particular technique, such as dispersant application, is required, the NEBA discussion provides an opportunity for that technique to be evaluated and pre-approved for spill situations matching the planning scenarios. Should a spill occur, stakeholders only need to verify that the assumptions considered in the NEBA and the pre-approval are still applicable.

## Conclusions

A total of 19 oil spill simulations corresponding to 19 different locations, were combined with bathymetric, meteorological, oceanographic, and geomorphological data in this paper to conclude:

- Oil slick trajectories in the Eastern Mediterranean are related to the seabed morphology. This is because strong seabed irregularities, present in the study area, are able to alter the local hydrodynamic regime.
- Oil spill modelling suggests the most vulnerable areas to be the eastern part of the Levantine Basin, offshore Israel and Lebanon, the Cyprus shorelines and the Egyptian coastline.
- Shoreline susceptibility varies significantly depending on differences in its morphology, degree of exposure to wave action, on the existence of uplifted wave-cut platforms, coastal lagoons and pools and the presence of tourist and environmentally sensitive zones.

This paper defends that key economic developments in the Eastern Mediterranean make oil spill models more crucial to spill monitoring and mitigation, and new legislative changes have recently made the interaction between Eastern Mediterranean countries more urgent than before. Recent hydrocarbon discoveries make the use of oil pollution dispersion models more than a necessity. Oil companies, licensed by the Republic of Cyprus to develop testing drills, are obliged to complete an Environmental Impact Study (EIA) according to national legislation. Oil spill dispersion models are part of this EIA. The EIAs are submitted to the Environmental Committee for approval before the execution of any testing drill. In addition, Israel has in October 2014 entered into the 2002 Emergency Protocol which accounts for prevention beyond the combating of oil pollution. Based on the results of this paper, we suggest the early deployment of coastal protection teams, following established protocols, in large oil spills accidents. Due to the significant environmental, social and economic impacts even a small oil spill can bring to the Eastern Mediterranean Sea, continuous improvement of the oil prevention and response capabilities is necessary. This can be achieved through investing in monitoring assets, technological innovation and forecasting models.

## Additional Information

**How to cite this article**: Alves, T. M. *et al*. Multidisciplinary oil spill modeling to protect coastal communities and the environment of the Eastern Mediterranean Sea. *Sci. Rep*. **6**, 36882; doi: 10.1038/srep36882 (2016).

**Publisher's note:** Springer Nature remains neutral with regard to jurisdictional claims in published maps and institutional affiliations.

## Supplementary Material

Supplementary Information

Supplementary Information

Supplementary Information

## References

[b1] Ferraro G. . Long term monitoring of oil spills in European seas. Int. J. Rem. Sensing 30, 627–645 (2009).

[b2] United Nations, Department of Economic and Social Affairs, Population Division. World Population Prospects: The 2015 Revision, Key Findings and Advance Tables. Working Paper No. ESA/P/WP.241 (2015).

[b3] Goldman R., Biton E., Brokovich E., Kark S. & Levin N. Oil spill contamination probability in the southeastern Levantine basin. Mar. Poll. Bull . 91, 347–356 (2015). 10.1016/j.marpolbul.2014.10.050 25534630

[b4] Guarrera L. Prospects of the Refining Sector in the Mediterranean Region. *19th World Petroleum Congress*, 29 June-3 July, Madrid, Spain, 23 pp. (2008).

[b5] Alves T. M., Kokinou E. & Zodiatis G. A three-step model to assess shoreline and offshore susceptibility to oil spills: The South Aegean (Crete) as an analogue for confined marine basins. Mar. Poll. Bull . 86, 443–457 (2014). 10.1016/j.marpolbul.2014.06.034 25113103

[b6] Robinson A. R. . General circulation of the Eastern Mediterranean. Earth-Sci. Rev. 32, 285–309 (1992).

[b7] Menna M., Poulain P.-M., Zodiatis G. & Gertman I. On the surface circulation of the Levantine sub-basin derived from Lagrangian drifters and satellite altimetry data. Deep Sea Research Part I: Oceanographic Research Papers 65, 46–58 (2012).

[b8] Brenner S. Structure and evolution of warm core eddies in the eastern Mediterranean Levantine Basin. J. Geoph. Res. 94, 12593–12602 (1989).

[b9] Zodiatis G., Drakopoulos P., Brenner S. & Groom S. Variability of the Cyprus warm core Eddy during the CYCLOPS project. Deep-Sea Res. II 52, 2897–2910 (2005).

[b10] Gertman I., Godman R., Ozer T. & Zodiatis G. Interannual Changes in the Thermohaline Structure of the south eastern Mediterranean. Rapp. Comm. Int. Mer Medit. 40, 211 (2013).

[b11] Groom S. . Satellite-derived spatial and temporal biological variability in the Cyprus Eddy. Deep-Sea Res. II 52, 2990–3010 (2005).

[b12] Alves T. M. . Modelling of oil spills in confined maritime basins: The case for early response in the Eastern Mediterranean Sea. Env. Poll . 206, 390–399 (2015). 10.1016/j.envpol.2015.07.042 26253313

[b13] Pinardi N., Arneri E., Crise A. & Ravaioli M. The physical, sedimentary and ecological structure and variability of shelf areas in the Mediterranean Sea. The Sea 14, 1243–1330 (2006).

[b14] Zodiatis G., Lardner R., Solovyov D., Panayidou X. & De Dominicis M. Predictions of oil slicks detected from satellite images using My Ocean forecasting data. Oc. Science 8, 1105–1115 (2012).

[b15] Coppini G. . Hindcast of oil-spill pollution during the Lebanon crisis in the Eastern Mediterranean, July–August 2006. Mar. Poll. Bull. 62, 140–153 (2011). 10.1016/j.marpolbul.2010.08.021 20880556

[b16] European Environmental Agency E15. Accidental oil spills from marine shipping. *Indic. Eur. Environ. Agency* E15 6 (2003) Date of access: 30/07/2016.

[b17] Marshall D. Influence of topography on the large-scale ocean circulation. J. Phys. Ocean . 25, 1622–1635 (1995).

[b18] Whitehead J. A. Topographic control of oceanic flows in deep passages and straits. Rev. Geophys. 36, 423–440 (1998).

[b19] Gille S. T., Metzger E. J. & Tokmakian R. Topography and ocean circulation. Oceanography 17, 47–54 (2004).

[b20] Lardner R. & Zodiatis G. An operational oil spill model in the Levantine Basin (Eastern Mediterranean Sea). Intern. Symp. Mar. Poll. 10, 5–9 (1998).

[b21] REMPEC. Workshop on Regional Response Capacity and Co-ordination for Major Oil Spill in the Mediterranean Sea (MEDEXPOL 2013). http://www.rempec.org/rempecnews.asp?NewsID=278 (2013). Date of access: 30/07/2016.

[b22] Panagiotakis C. & Kokinou E. Linear Pattern Detection of Geological Faults via a Topology and Shape Optimization Method. IEEE J. Sel. Top. Appl. Earth Obs. Rem. Sens . 8, 3–11 (2015).

[b23] Kokinou E. Geomorphologic features of the marine environment in Eastern Mediterranean using a modern processing approach. (ed. Schaeben H., Delgado R. T., van den Boogaart G., van den Boogaart R. ), Proceedings of IAMG 2015, The 17th Annual Conference of the International Association for Mathematical Geosciences, 436–445 (2015).

[b24] Berthou P. . EMODNET-The European Marine Observation and Data Network. Eur. Sci. Found.–Mar. Board 10 (2008).

[b25] Adler E. & Inbar M. Shoreline sensitivity to oil spills, the Mediterranean coast of Israel: Assessment and analysis. Ocean & Coast. Manag. 50, 24–34 (2007).

[b26] Palomino D., Vázquez J.-T., Ercilla G., Alonso B., López-González N. & Díaz-del-Río V. Interaction between seabed morphology and water masses around the seamounts on the Motril Marginal Plateau (Alboran Sea, Western Mediterranean). Geo-Marine Letters 31(5–6), 465–479 (2011).

[b27] Zodiatis G., Hayes D., Gertman I. & Samuel-Rhodes Y. The Cyprus warm eddy and the Atlantic water during the CYBO cruises (1995-2009). Rapp. Comm. Intern. Mer Meditter. 39, 202 pp (2010).

[b28] Zodiatis G. . On the main flow features of the SE Levantine (CYBO cruises 1995-2012). Geophys. Res. Abst. 15, EGU2013-9861 (2013).

[b29] Zodiatis G. . Modeling oil spills in the Med-Sea as a mean of early response in cases of oil leakages. EGU Gen. Ass. 18, EGU2016-14174 (2016).

[b30] SAFEMED. Study of Maritime Traffic Flows in the Mediterranean Sea. MEDA Regional Project 2005/109-573 , 40pp (2008).

[b31] UNEP. Lebanon Post-Conflict Environmental Assessment. ISBN: 978-92-807-2794-4 http://postconflict.unep.ch/publications.php?prog=lebanon (2007) Date of access: 30/07/2016.

[b32] World Bank. Republic of Lebanon Economic Assessment of Environmental Degradation due to July 2006 Hostilities (2007).

