# Supplementary material for: Multidisciplinary oil spill modeling to protect coastal communities and the environment of the Eastern Mediterranean Sea
Source: Sci Rep. 2016 Nov 10;6:36882. doi: 10.1038/srep36882 (PMC5103274; doi:10.1038/srep36882)
Supplement: Supplementary Information [file srep36882-s1.pdf]

Multidisciplinary oil spill modeling to protect coastal communities and the environment of the Eastern Mediterranean Sea

Tiago M. Alves[1,*], Eleni Kokinou[2], George Zodiatis[3], Hari Radhakrishnan[3], Costas Panagiotakis[4] and Robin Lardner[3]

1) 3D Seismic Lab - School of Earth and Ocean Sciences, Cardiff University - Main Building, Park Place, Cardiff, CF10 3AT, UK (alvest@cardiff.ac.uk)
2) Department of Environmental and Natural Resources Engineering, Technological Educational Institute Crete, 3 Romanou Str. Chalepa, Chania, Crete GR 73133, Greece (ekokinou@staff.teicrete.gr)
3) Oceanography Centre, University of Cyprus, P.O. Box 20537, 1678 Nicosia, Cyprus (gzodiac@ucy.ac.cy)
4) Department of Business Administration, Technological Educational Institute Crete, Agios Nikolaos, Greece (cpanag@staff.teicrete.gr)

Oil Fate for Worst-Case Scenario: Site 01

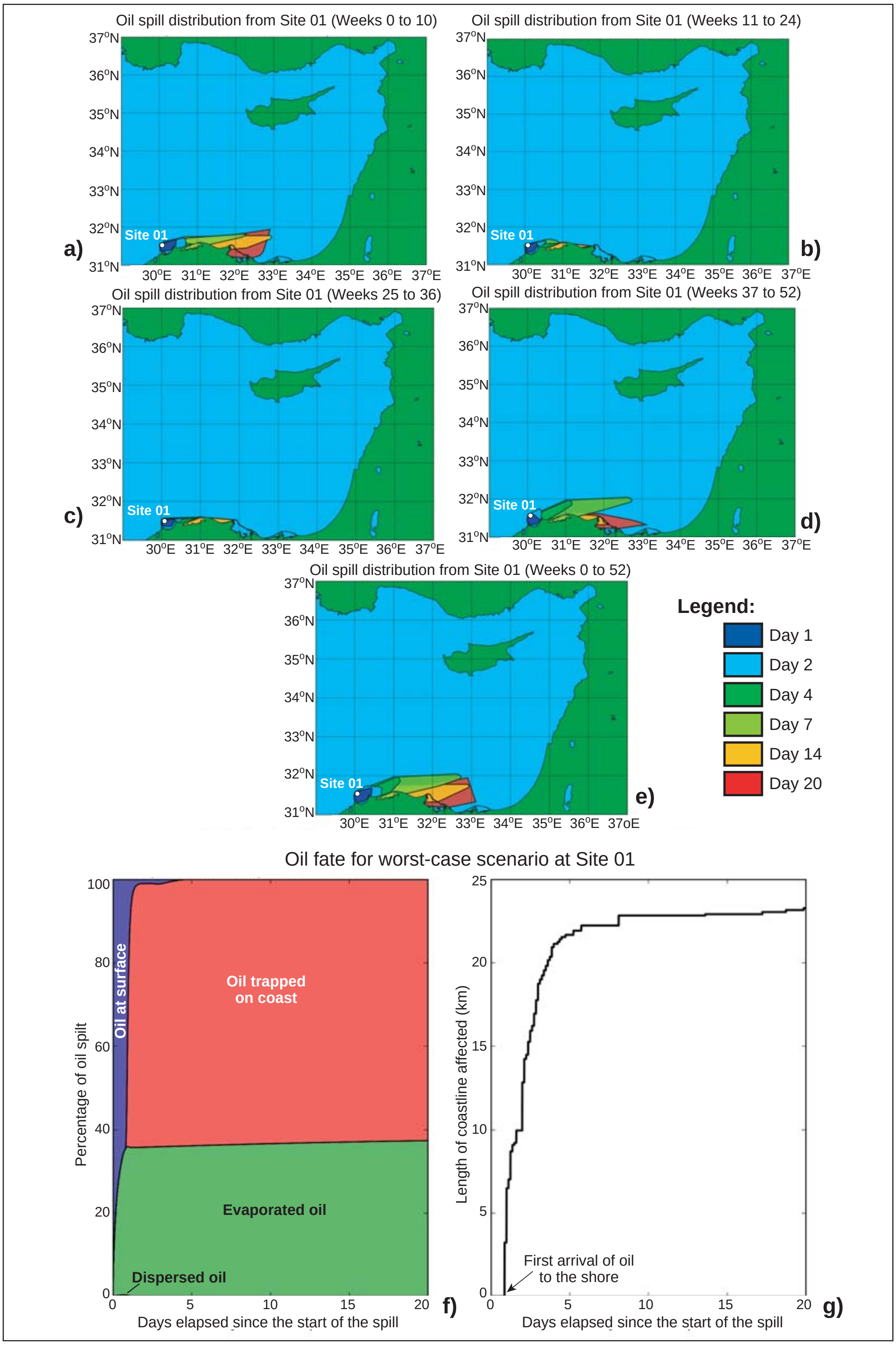

Supplementary Figure 1 - MEDSLIK model results for Site 01, which reflects an oil spill one (1) nautical mile off the port of Alexandria, Egypt. a) Oil spill distribution in weeks 0 to 10. b) Oil spill distribution in weeks 12 to 24. c) Oil spill distribution in weeks 25 to 36. d) Oil spill distribution in weeks 37 to 52. e) Oil spill distribution considering average meteorological and oceanographic conditions for weeks 0 to 50. f) Oil fate plot for days 0 to 20 after the oil spill. Yellow-displaced oil; Green-evaporated oil; Red-oil trapped on coast; Blue-oil at the surface. g) Plot showing the length of affected coast 0 to 20 days after the initial oil spill according to the MEDSLIK results. Figure was generated by the authors using MEDSLIK (http://oceanography.ucy.ac.cy/medslik/).

Multidisciplinary oil spill modeling to protect coastal communities and the environment of the Eastern Mediterranean Sea

Tiago M. Alves[1,*], Eleni Kokinou[2], George Zodiatis[3], Hari Radhakrishnan[3], Costas Panagiotakis[4] and Robin Lardner[3]

1) 3D Seismic Lab - School of Earth and Ocean Sciences, Cardiff University - Main Building, Park Place, Cardiff, CF10 3AT, UK (alvest@cardiff.ac.uk)
2) Department of Environmental and Natural Resources Engineering, Technological Educational Institute Crete, 3 Romanou Str. Chalepa, Chania, Crete GR 73133, Greece (ekokinou@staff.teicrete.gr)
3) Oceanography Centre, University of Cyprus, P.O. Box 20537, 1678 Nicosia, Cyprus (gzodiac@ucy.ac.cy)
4) Department of Business Administration, Technological Educational Institute Crete, Agios Nikolaos, Greece (cpanag@staff.teicrete.gr)

Oil Fate for Worst-Case Scenario: Site 02

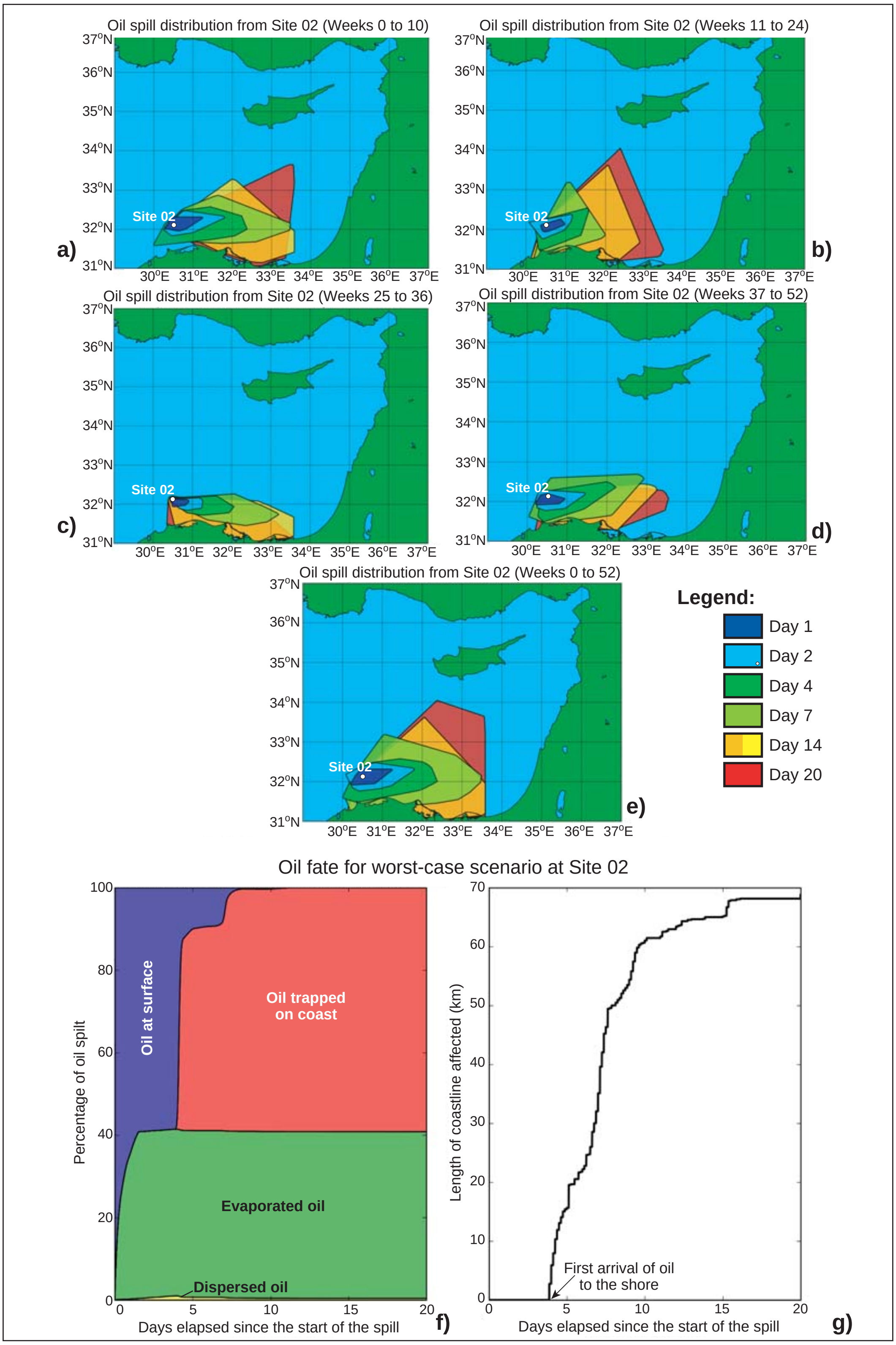

Supplementary Figure 2 - MEDSLIK model results for Site 02, located at a major oil and gas platform offshore the Idku port. a) Oil spill distribution in weeks 0 to 10. b) Oil spill distribution in weeks 12 to 24. c) Oil spill distribution in weeks 25 to 36. d) Oil spill distribution in weeks 37 to 52. e) Oil spill distribution considering average meteorological and oceanographic conditions for weeks 0 to 50. f) Oil fate plot for days 0 to 20 after the oil spill. Yellow-displaced oil; Green-evaporated oil; Red-oil trapped on coast; Blue-oil at the surface. g) Plot showing the length of affected coast 0 to 20 days after the initial oil spill according to the MEDSLIK results. Figure was generated by the authors using MEDSLIK (http://oceanography.ucy.ac.cy/medslik/).

Multidisciplinary oil spill modeling to protect coastal communities and the environment of the Eastern Mediterranean Sea

Tiago M. Alves[1,*], Eleni Kokinou[2], George Zodiatis[3], Hari Radhakrishnan[3], Costas Panagiotakis[4] and Robin Lardner[3]

1) 3D Seismic Lab - School of Earth and Ocean Sciences, Cardiff University - Main Building, Park Place, Cardiff, CF10 3AT, UK (alvest@cardiff.ac.uk)
2) Department of Environmental and Natural Resources Engineering, Technological Educational Institute Crete, 3 Romanou Str. Chalepa, Chania, Crete GR 73133, Greece (ekokinou@staff.teicrete.gr)
3) Oceanography Centre, University of Cyprus, P.O. Box 20537, 1678 Nicosia, Cyprus (gzodiac@ucy.ac.cy)
4) Department of Business Administration, Technological Educational Institute Crete, Agios Nikolaos, Greece (cpanag@staff.teicrete.gr)

Oil Fate for Worst-Case Scenario: Site 03

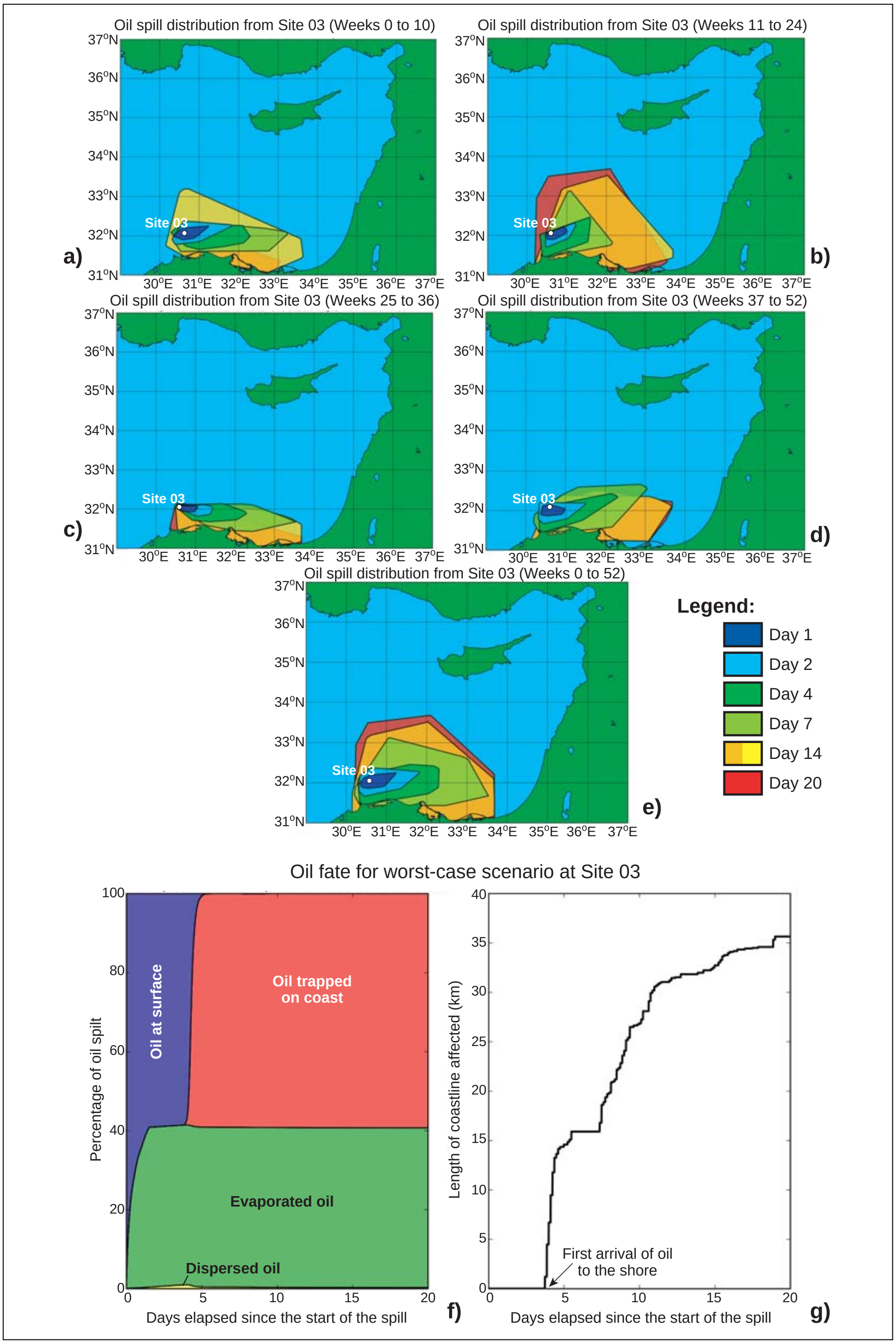

Supplementary Figure 3 - MEDSLIK model results for Site 03, located 20 nautical miles NW of the Nile River Mouth. a) Oil spill movement for weeks 0 to 10. b) Oil spill movement for weeks 12 to 24. c) Oil spill movement for weeks 26 to 36. d) Oil spill movement for weeks 38 to 50. e) Oil spill movement considering average meterological and oceanographic conditions for weeks 0 to 50. f) Oil fate plot for days 0 to 20 after the oil spill. Yellow-displaced oil; Green-evaporated oil; Red-oil trapped on coast; Blue-oil at the surface. g) Plot showing the length of affected coast 0 to 20 days after the initial oil spill according to the MEDSLIK results. Figure was generated by the authors using MEDSLIK (http://oceanography.ucy.ac.cy/medslik/).

Multidisciplinary oil spill modeling to protect coastal communities and the environment of the Eastern Mediterranean Sea

Tiago M. Alves[1,*], Eleni Kokinou[2], George Zodiatis[3], Hari Radhakrishnan[3], Costas Panagiotakis[4] and Robin Lardner[3]

1) 3D Seismic Lab - School of Earth and Ocean Sciences, Cardiff University - Main Building, Park Place, Cardiff, CF10 3AT, UK (alvest@cardiff.ac.uk)
2) Department of Environmental and Natural Resources Engineering, Technological Educational Institute Crete, 3 Romanou Str. Chalepa, Chania, Crete GR 73133, Greece (ekokinou@staff.teicrete.gr)
3) Oceanography Centre, University of Cyprus, P.O. Box 20537, 1678 Nicosia, Cyprus (gzodiac@ucy.ac.cy)
4) Department of Business Administration, Technological Educational Institute Crete, Agios Nikolaos, Greece (cpanag@staff.teicrete.gr)

Oil Fate for Worst-Case Scenario: Site 04

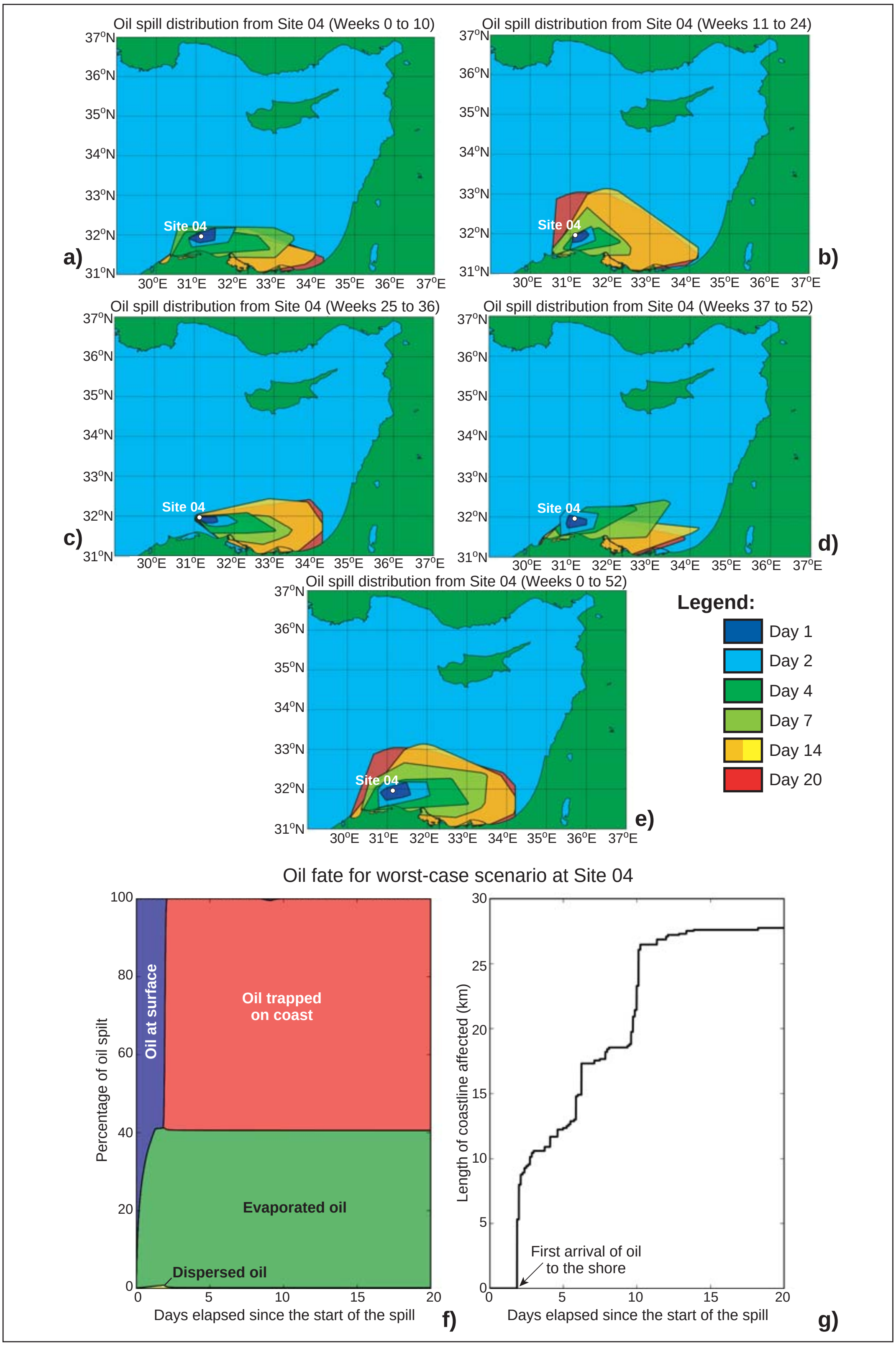

Supplementary Figure 4 - MEDSLIK model results for Site 04, located at a major oil and gas platform offshore the Nile Delta. a) Oil spill distribution in weeks 0 to 10. b) Oil spill distribution in weeks 12 to 24. c) Oil spill distribution in weeks 25 to 36. d) Oil spill distribution in weeks 37 to 52. e) Oil spill distribution considering average meteorological and oceanographic conditions for weeks 0 to 50. f) Oil fate plot for days 0 to 20 after the oil spill. Yellow-displaced oil; Green-evaporated oil; Red-oil trapped on coast; Blue-oil at the surface. g) Plot showing the length of affected coast 0 to 20 days after the initial oil spill according to the MEDSLIK results. Figure was generated by the authors using MEDSLIK (http://oceanography.ucy.ac.cy/medslik/).

Multidisciplinary oil spill modeling to protect coastal communities and the environment of the Eastern Mediterranean Sea

Tiago M. Alves[1,*], Eleni Kokinou[2], George Zodiatis[3], Hari Radhakrishnan[3], Costas Panagiotakis[4] and Robin Lardner[3]

1) 3D Seismic Lab - School of Earth and Ocean Sciences, Cardiff University - Main Building, Park Place, Cardiff, CF10 3AT, UK (alvest@cardiff.ac.uk)
2) Department of Environmental and Natural Resources Engineering, Technological Educational Institute Crete, 3 Romanou Str. Chalepa, Chania, Crete GR 73133, Greece (ekokinou@staff.teicrete.gr)
3) Oceanography Centre, University of Cyprus, P.O. Box 20537, 1678 Nicosia, Cyprus (gzodiac@ucy.ac.cy)
4) Department of Business Administration, Technological Educational Institute Crete, Agios Nikolaos, Greece (cpanag@staff.teicrete.gr)

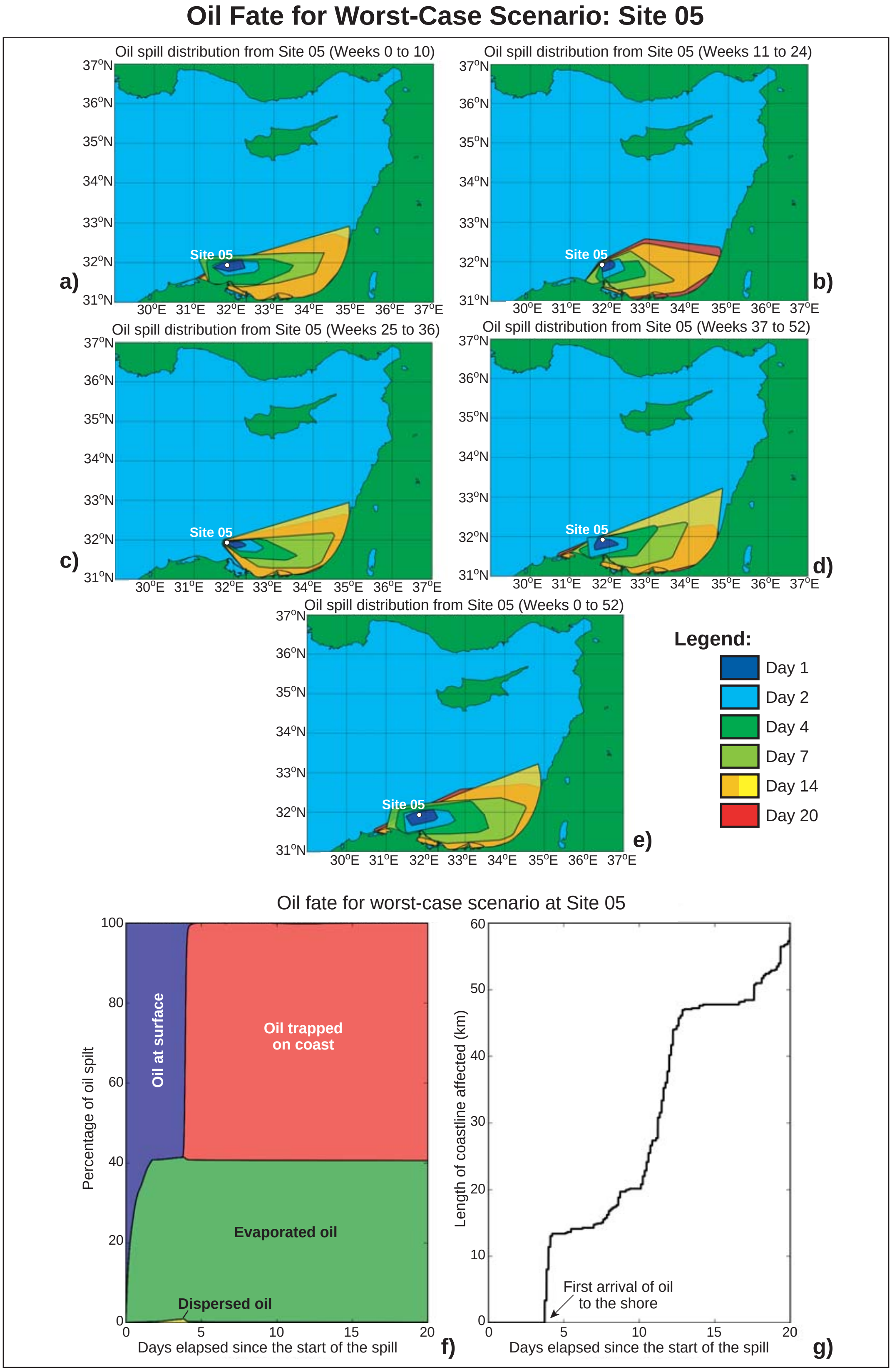

Supplementary Figure 5 - MEDSLIK model results for Site 05, located 20 nautical miles North of the Damietta River Mouth. a) Oil spill distribution in weeks 0 to 10. b) Oil spill distribution in weeks 12 to 24. c) Oil spill distribution in weeks 25 to 36. d) Oil spill distribution in weeks 37 to 52. e) Oil spill distribution considering average meteorological and oceanographic conditions for weeks 0 to 50. f) Oil fate plot for days 0 to 20 after the oil spill. Yellow-displaced oil; Green-evaporated oil; Red-oil trapped on coast; Blue-oil at the surface. g) Plot showing the length of affected coast 0 to 20 days after the initial oil spill according to the MEDSLIK results. Figure was generated by the authors using MEDSLIK (http://oceanography.ucy.ac.cy/medslik/).

Tiago M. Alves[1,*], Eleni Kokinou[2], George Zodiatis[3], Hari Radhakrishnan[3], Costas Panagiotakis[4] and Robin Lardner[3]

1) 3D Seismic Lab - School of Earth and Ocean Sciences, Cardiff University - Main Building, Park Place, Cardiff, CF10 3AT, UK (alvest@cardiff.ac.uk)
2) Department of Environmental and Natural Resources Engineering, Technological Educational Institute Crete, 3 Romanou Str. Chalepa, Chania, Crete GR 73133, Greece (ekokinou@staff.teicrete.gr)
3) Oceanography Centre, University of Cyprus, P.O. Box 20537, 1678 Nicosia, Cyprus (gzodiac@ucy.ac.cy)
4) Department of Business Administration, Technological Educational Institute Crete, Agios Nikolaos, Greece (cpanag@staff.teicrete.gr)

Oil Fate for Worst-Case Scenario: Site 06

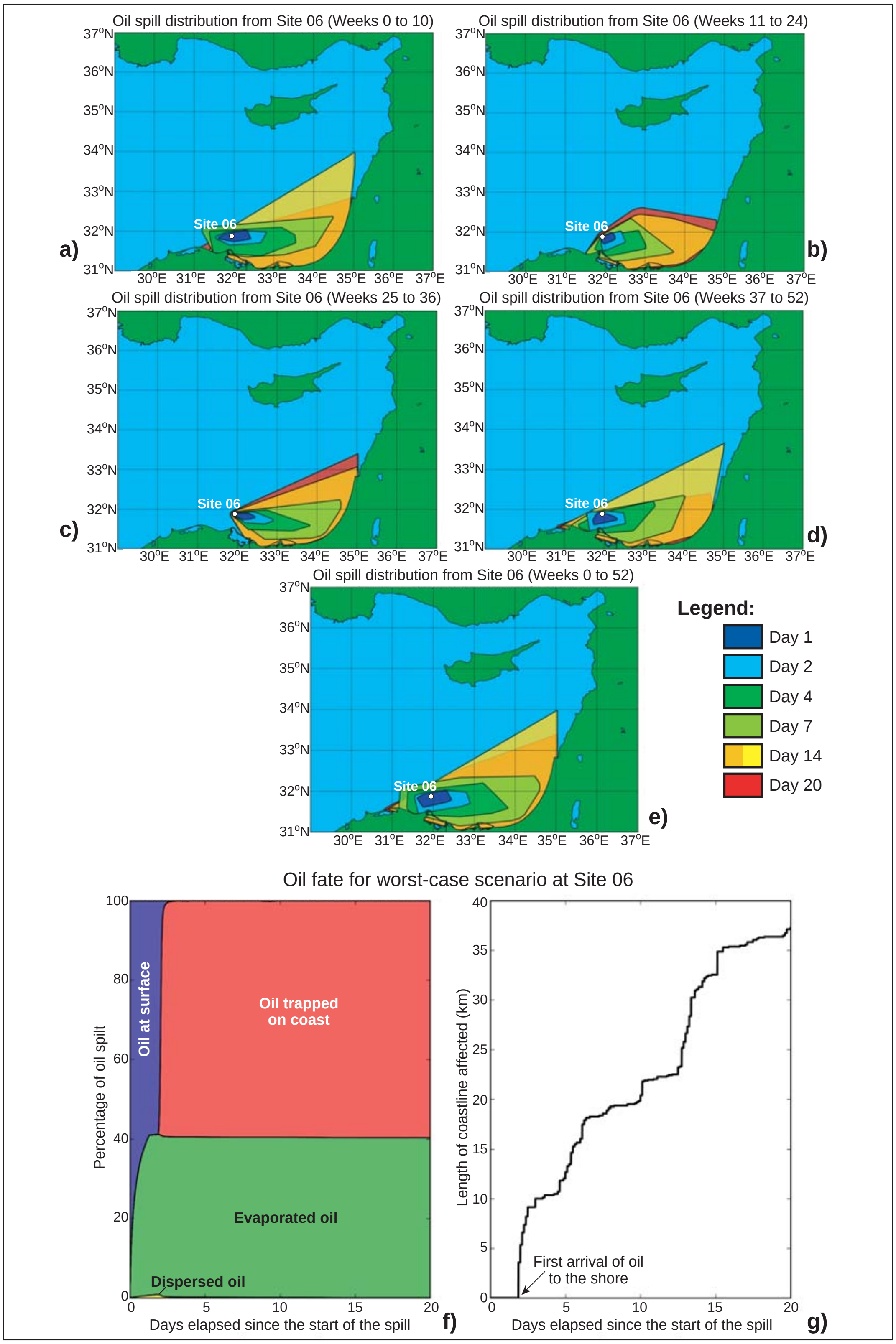

Supplementary Figure 6 - MEDSLIK model results for Site 06, located at an oil and gas platform approximately 20 nautical miles NE of the Damietta River Mouth. a) Oil spill distribution in weeks 0 to 10. b) Oil spill distribution in weeks 12 to 24. c) Oil spill distribution in weeks 25 to 36. d) Oil spill distribution in weeks 37 to 52. e) Oil spill distribution considering average meteorological and oceanographic conditions for weeks 0 to 50. f) Oil fate plot for days 0 to 20 after the oil spill. Yellow-displaced oil; Green-evaporated oil; Red-oil trapped on coast; Blue-oil at the surface. g) Plot showing the length of affected coast 0 to 20 days after the initial oil spill according to the MEDSLIK results. Figure was generated by the authors using MEDSLIK (http://oceanography.ucy.ac.cy/medslik/).

Multidisciplinary oil spill modeling to protect coastal communities and the environment of the Eastern Mediterranean Sea

Tiago M. Alves[1,*], Eleni Kokinou[2], George Zodiatis[3], Hari Radhakrishnan[3], Costas Panagiotakis[4] and Robin Lardner[3]

1) 3D Seismic Lab - School of Earth and Ocean Sciences, Cardiff University - Main Building, Park Place, Cardiff, CF10 3AT, UK (alvest@cardiff.ac.uk)
2) Department of Environmental and Natural Resources Engineering, Technological Educational Institute Crete, 3 Romanou Str. Chalepa, Chania, Crete GR 73133, Greece (ekokinou@staff.teicrete.gr)
3) Oceanography Centre, University of Cyprus, P.O. Box 20537, 1678 Nicosia, Cyprus (gzodiac@ucy.ac.cy)
4) Department of Business Administration, Technological Educational Institute Crete, Agios Nikolaos, Greece (cpanag@staff.teicrete.gr)

Oil Fate for Worst-Case Scenario: Site 07

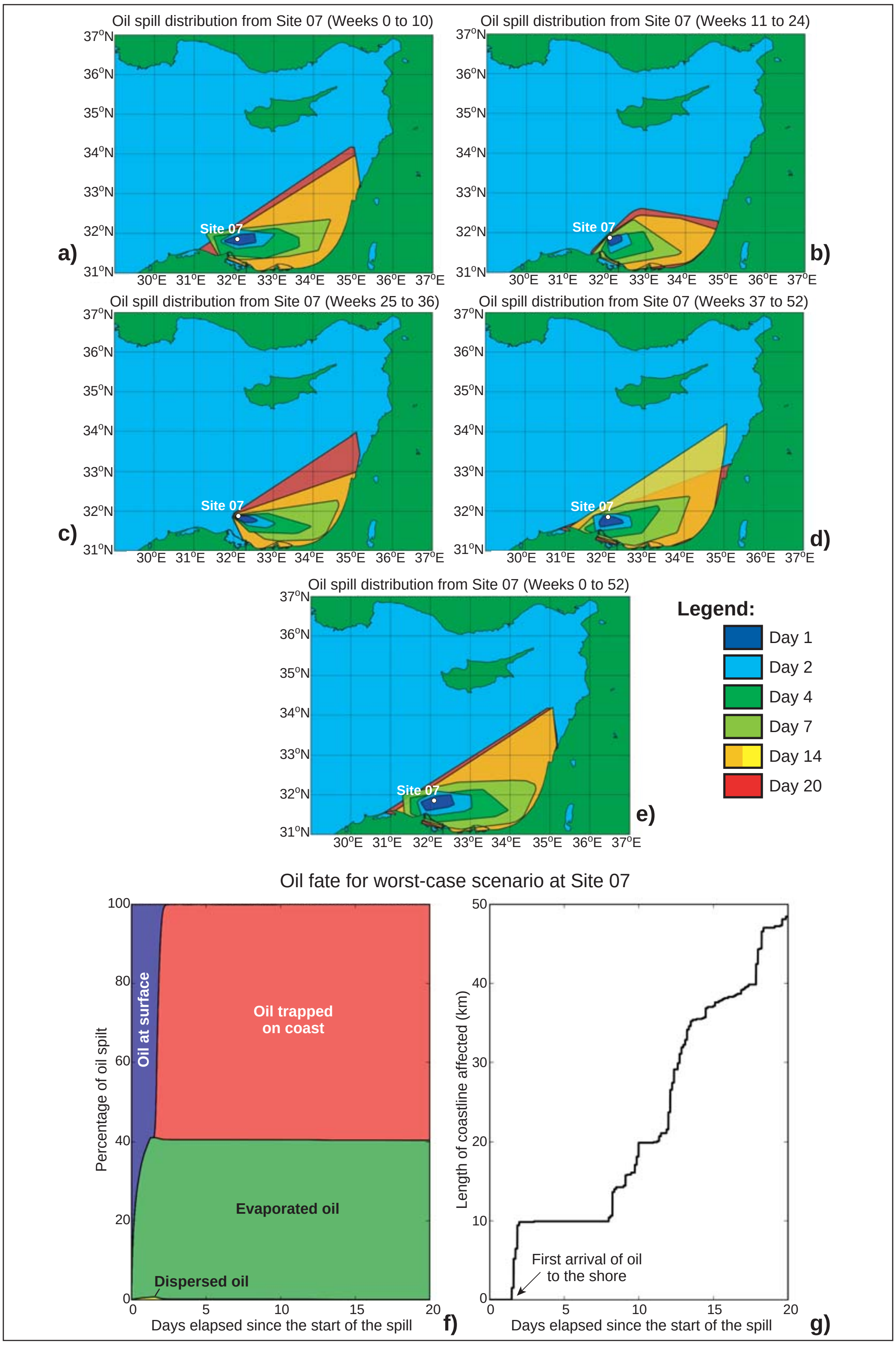

Supplementary Figure 7 - MEDSLIK model results for Site 07, located 20 nautical miles NE of the Damietta River Mouth at a major oil and gas platform. a) Oil spill distribution in weeks 0 to 10. b) Oil spill distribution in weeks 12 to 24. c) Oil spill distribution in weeks 25 to 36. d) Oil spill distribution in weeks 37 to 52. e) Oil spill distribution considering average meteorological and oceanographic conditions for weeks 0 to 50. f) Oil fate plot for days 0 to 20 after the oil spill. Yellow-displaced oil; Green-evaporated oil; Red-oil trapped on coast; Blue-oil at the surface. g) Plot showing the length of affected coast 0 to 20 days after the initial oil spill according to the MEDSLIK results. Figure was generated by the authors using MEDSLIK (http://oceanography.ucy.ac.cy/medslik/).

Multidisciplinary oil spill modeling to protect coastal communities and the environment of the Eastern Mediterranean Sea

Tiago M. Alves[1,*], Eleni Kokinou[2], George Zodiatis[3], Hari Radhakrishnan[3], Costas Panagiotakis[4] and Robin Lardner[3]

1) 3D Seismic Lab - School of Earth and Ocean Sciences, Cardiff University - Main Building, Park Place, Cardiff, CF10 3AT, UK (alvest@cardiff.ac.uk)
2) Department of Environmental and Natural Resources Engineering, Technological Educational Institute Crete, 3 Romanou Str. Chalepa, Chania, Crete GR 73133, Greece (ekokinou@staff.teicrete.gr)
3) Oceanography Centre, University of Cyprus, P.O. Box 20537, 1678 Nicosia, Cyprus (gzodiac@ucy.ac.cy)
4) Department of Business Administration, Technological Educational Institute Crete, Agios Nikolaos, Greece (cpanag@staff.teicrete.gr)

Oil Fate for Worst-Case Scenario: Site 08

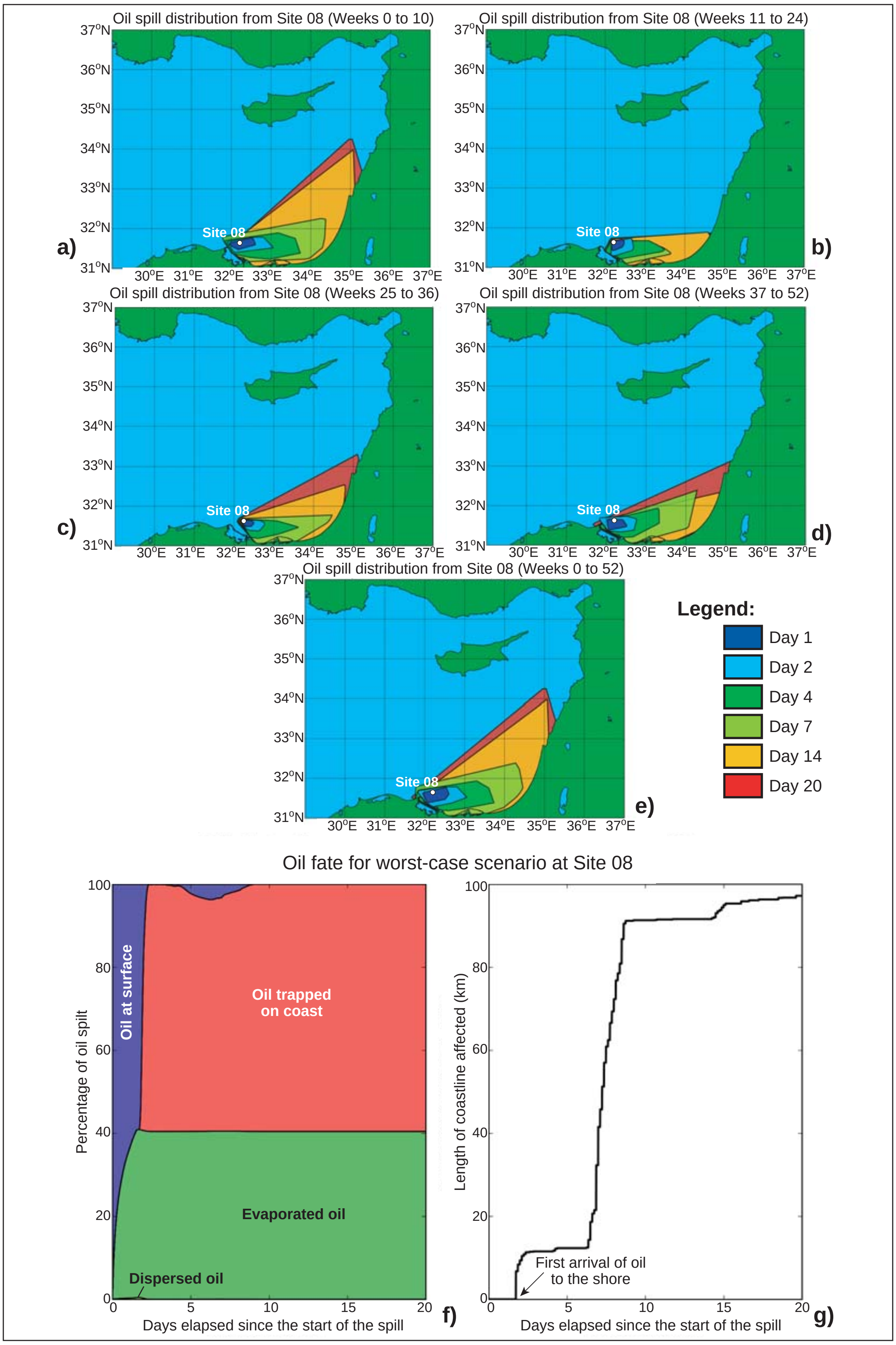

Supplementary Figure 8 - MEDSLIK model results for Site 08, located approximately 15 nautical miles North of the Suez Canal. a) Oil spill distribution in weeks 0 to 10. b) Oil spill distribution in weeks 12 to 24. c) Oil spill distribution in weeks 25 to 36. d) Oil spill distribution in weeks 37 to 52. e) Oil spill distribution considering average meteorological and oceanographic conditions for weeks 0 to 50. f) Oil fate plot for days 0 to 20 after the oil spill. Yellow-displaced oil; Green-evaporated oil; Red-oil trapped on coast; Blue-oil at the surface. g) Plot showing the length of affected coast 0 to 20 days after the initial oil spill according to the MEDSLIK results. Figure was generated by the authors using MEDSLIK (http://oceanography.ucy.ac.cy/medslik/).

Multidisciplinary oil spill modeling to protect coastal communities and the environment of the Eastern Mediterranean Sea

Tiago M. Alves[1,*], Eleni Kokinou[2], George Zodiatis[3], Hari Radhakrishnan[3], Costas Panagiotakis[4] and Robin Lardner[3]

1) 3D Seismic Lab - School of Earth and Ocean Sciences, Cardiff University - Main Building, Park Place, Cardiff, CF10 3AT, UK (alvest@cardiff.ac.uk)
2) Department of Environmental and Natural Resources Engineering, Technological Educational Institute Crete, 3 Romanou Str. Chalepa, Chania, Crete GR 73133, Greece (ekokinou@staff.teicrete.gr)
3) Oceanography Centre, University of Cyprus, P.O. Box 20537, 1678 Nicosia, Cyprus (gzodiac@ucy.ac.cy)
4) Department of Business Administration, Technological Educational Institute Crete, Agios Nikolaos, Greece (cpanag@staff.teicrete.gr)

Oil Fate for Worst-Case Scenario: Site 09

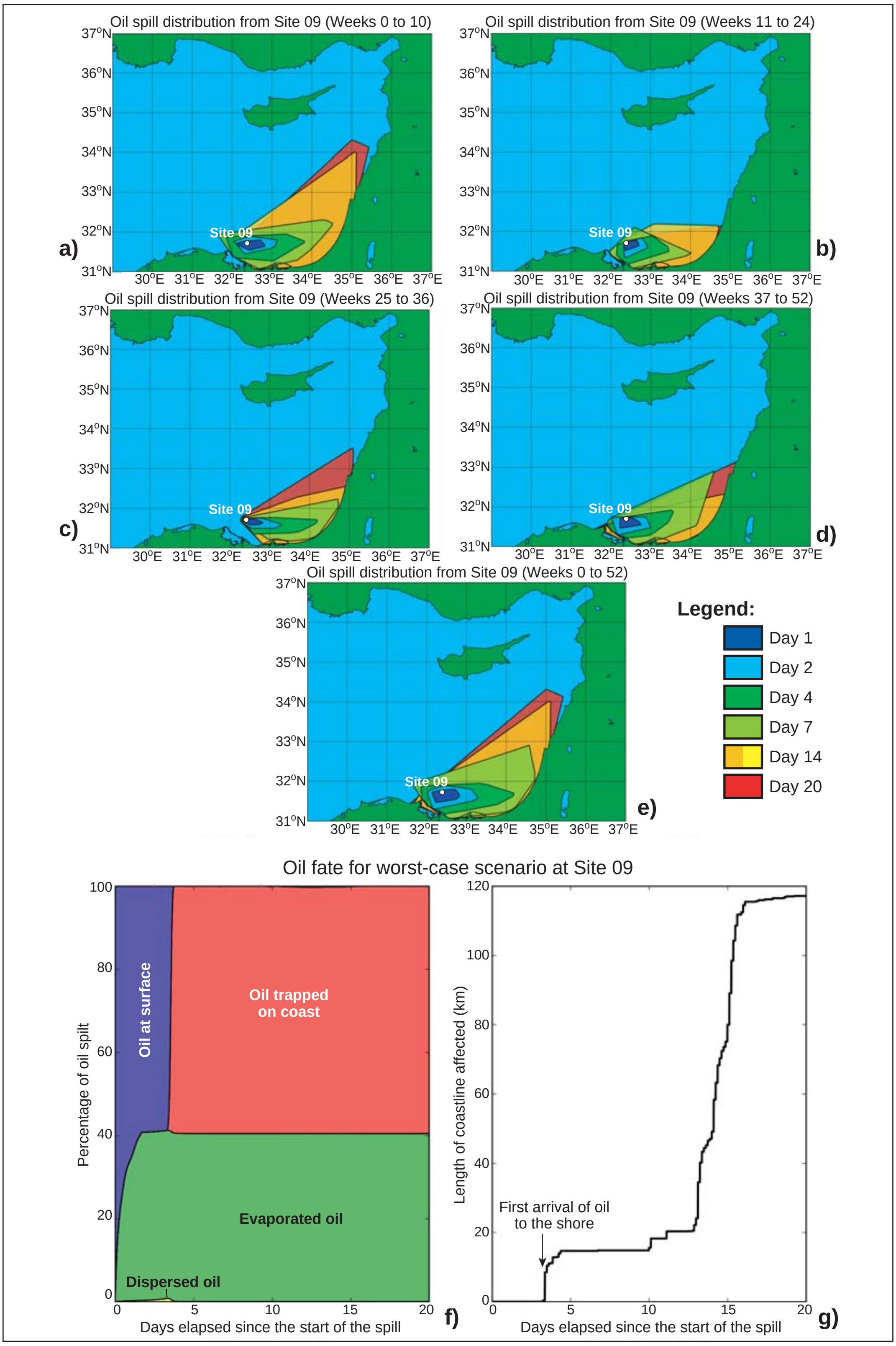

Supplementary Figure 9 - MEDSLIK model results for Site 09, corresponding to an accident located 25 miles North of the Suez Canal. a) Oil spill distribution in weeks 0 to 10. b) Oil spill distribution in weeks 12 to 24. c) Oil spill distribution in weeks 25 to 36. d) Oil spill distribution in weeks 37 to 52. e) Oil spill distribution considering average meteorological and oceanographic conditions for weeks 0 to 50. f) Oil fate plot for days 0 to 20 after the oil spill. Yellow-displaced oil; Green-evaporated oil; Red-oil trapped on coast; Blue-oil at the surface. g) Plot showing the length of affected coast 0 to 20 days after the initial oil spill according to the MEDSLIK results. Figure was generated by the authors using MEDSLIK (http://oceanography.ucy.ac.cy/medslik/).

Multidisciplinary oil spill modeling to protect coastal communities and the environment of the Eastern Mediterranean Sea

Tiago M. Alves[1,*], Eleni Kokinou[2], George Zodiatis[3], Hari Radhakrishnan[3], Costas Panagiotakis[4] and Robin Lardner[3]

1) 3D Seismic Lab - School of Earth and Ocean Sciences, Cardiff University - Main Building, Park Place, Cardiff, CF10 3AT, UK (alvest@cardiff.ac.uk)
2) Department of Environmental and Natural Resources Engineering, Technological Educational Institute Crete, 3 Romanou Str. Chalepa, Chania, Crete GR 73133, Greece (ekokinou@staff.teicrete.gr)
3) Oceanography Centre, University of Cyprus, P.O. Box 20537, 1678 Nicosia, Cyprus (gzodiac@ucy.ac.cy)
4) Department of Business Administration, Technological Educational Institute Crete, Agios Nikolaos, Greece (cpanag@staff.teicrete.gr)

Oil Fate for Worst-Case Scenario: Site 10

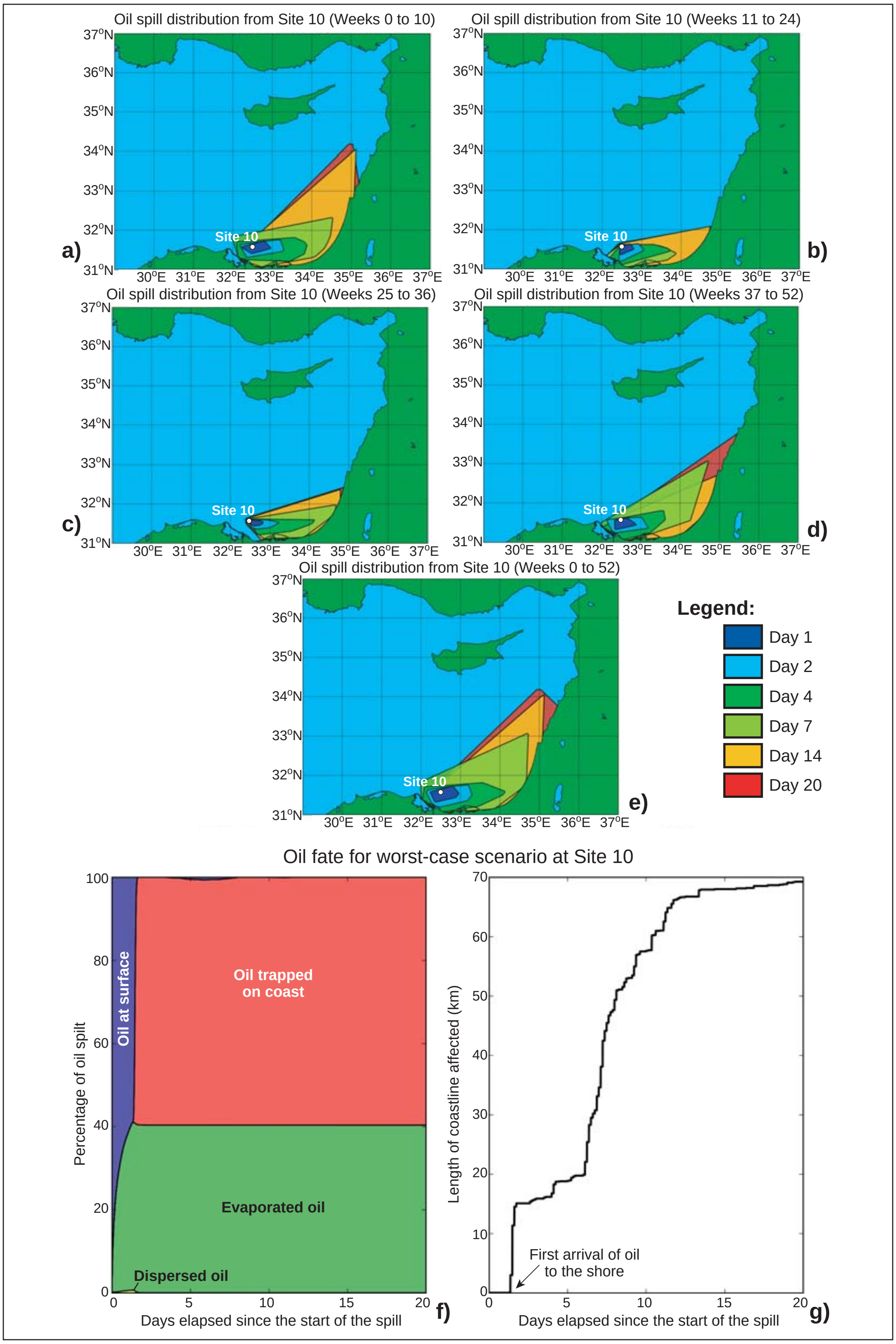

Supplementary Figure 10 - MEDSLIK model results for Site 10, located 15 nautical miles NE of the Suez Canal. a) Oil spill distribution in weeks 0 to 10. b) Oil spill distribution in weeks 12 to 24. c) Oil spill distribution in weeks 25 to 36. d) Oil spill distribution in weeks 37 to 52. e) Oil spill distribution considering average meteorological and oceanographic conditions for weeks 0 to 50. f) Oil fate plot for days 0 to 20 after the oil spill. Yellow-displaced oil; Green-evaporated oil; Red-oil trapped on coast; Blue-oil at the surface. g) Plot showing the length of affected coast 0 to 20 days after the initial oil spill according to the MEDSLIK results. Figure was generated by the authors using MEDSLIK (http://oceanography.ucy.ac.cy/medslik/).

Multidisciplinary oil spill modeling to protect coastal communities and the environment of the Eastern Mediterranean Sea

Tiago M. Alves[1,*], Eleni Kokinou[2], George Zodiatis[3], Hari Radhakrishnan[3], Costas Panagiotakis[4] and Robin Lardner[3]

1) 3D Seismic Lab - School of Earth and Ocean Sciences, Cardiff University - Main Building, Park Place, Cardiff, CF10 3AT, UK (alvest@cardiff.ac.uk)
2) Department of Environmental and Natural Resources Engineering, Technological Educational Institute Crete, 3 Romanou Str. Chalepa, Chania, Crete GR 73133, Greece (ekokinou@staff.teicrete.gr)
3) Oceanography Centre, University of Cyprus, P.O. Box 20537, 1678 Nicosia, Cyprus (gzodiac@ucy.ac.cy)
4) Department of Business Administration, Technological Educational Institute Crete, Agios Nikolaos, Greece (cpanag@staff.teicrete.gr)

Oil Fate for Worst-Case Scenario: Site 11

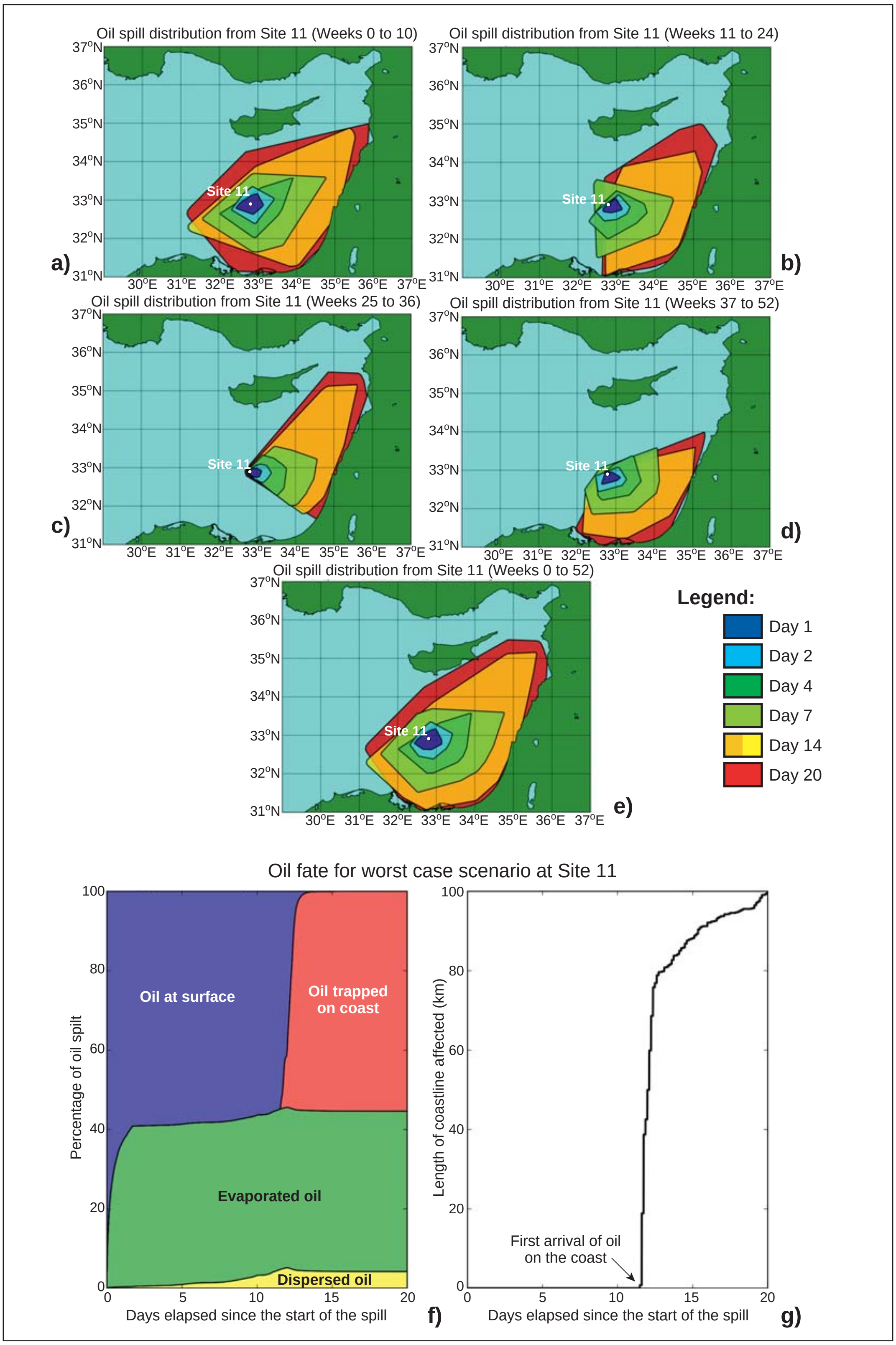

Supplementary Figure 11 - MEDSLIK model results for Site 11 located close to the Zhor field, offshore Egypt. a) Oil spill movement for weeks 0 to 10. b) Oil spill movement for weeks 11 to 24. c) Oil spill movement for weeks 25 to 36. d) Oil spill movement for weeks 37 to 52. e) Oil spill movement considering average meterological and oceanographic conditions for weeks 0 to 52. f) Oil fate plot for days 0 to 20 after the oil spill. Yellow-displaced oil; Green-evaporated oil; Red-oil trapped on coast; Blue-oil at the surface. g) Plot showing the length of affected coast 0 to 20 days after the initial oil spill according to the MEDSLIK results. Figure was generated by the authors using MEDSLIK (http://oceanography.ucy.ac.cy/medslik/).

Multidisciplinary oil spill modeling to protect coastal communities and the environment of the Eastern Mediterranean Sea

Tiago M. Alves[1,*], Eleni Kokinou[2], George Zodiatis[3], Hari Radhakrishnan[3], Costas Panagiotakis[4] and Robin Lardner[3]

1) 3D Seismic Lab - School of Earth and Ocean Sciences, Cardiff University - Main Building, Park Place, Cardiff, CF10 3AT, UK (alvest@cardiff.ac.uk)
2) Department of Environmental and Natural Resources Engineering, Technological Educational Institute Crete, 3 Romanou Str. Chalepa, Chania, Crete GR 73133, Greece (ekokinou@staff.teicrete.gr)
3) Oceanography Centre, University of Cyprus, P.O. Box 20537, 1678 Nicosia, Cyprus (gzodiac@ucy.ac.cy)
4) Department of Business Administration, Technological Educational Institute Crete, Agios Nikolaos, Greece (cpanag@staff.teicrete.gr)

Oil Fate for Worst-Case Scenario: Site 12

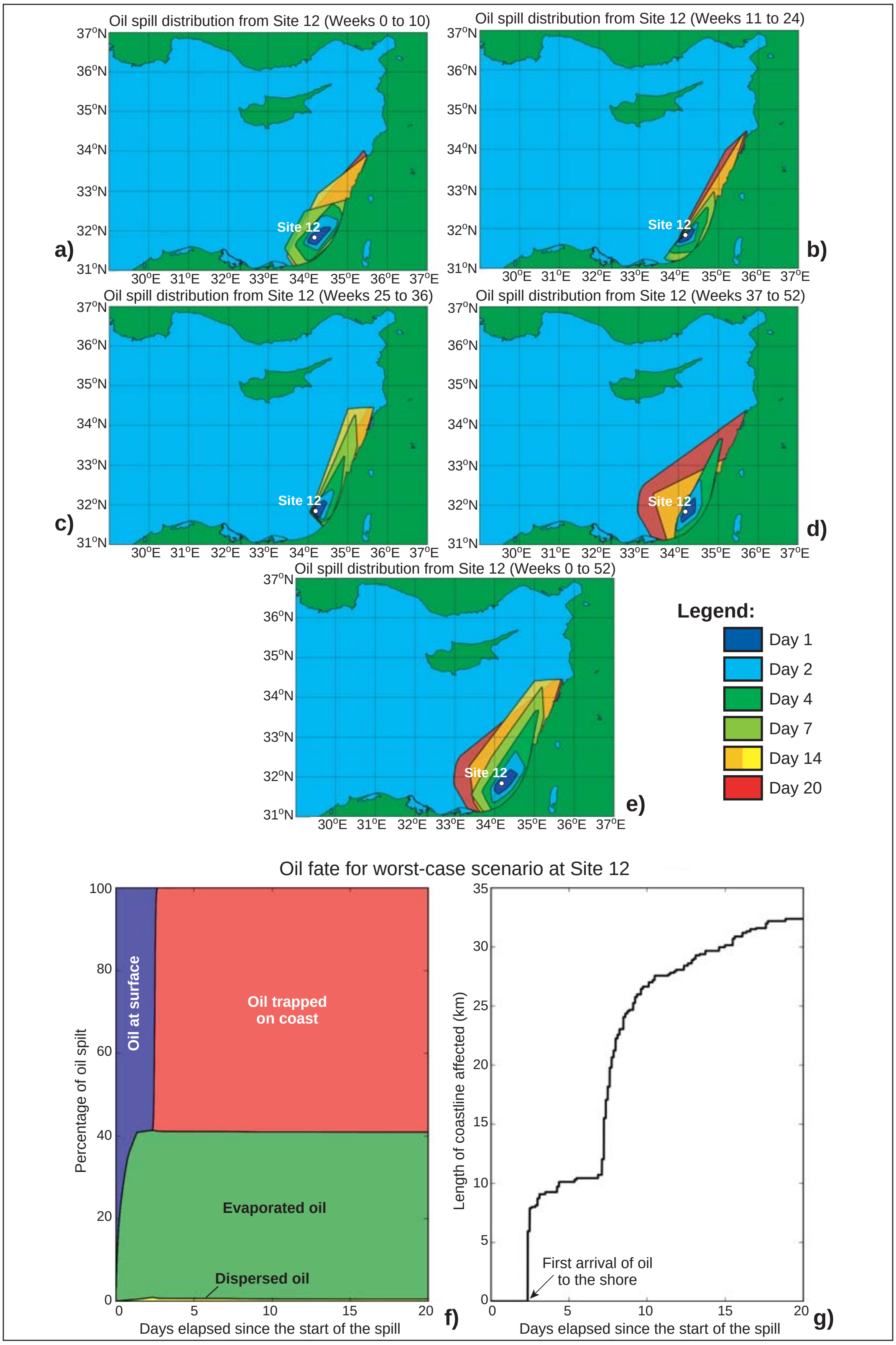

Supplementary Figure 12 - MEDSLIK model results for Site 12, located at the Marine-1 and Marine-2 oil and gas platforms offshore Israel. a) Oil spill distribution in weeks 0 to 10. b) Oil spill distribution in weeks 12 to 24. c) Oil spill distribution in weeks 25 to 36. d) Oil spill distribution in weeks 37 to 52. e) Oil spill distribution considering average meteorological and oceanographic conditions for weeks 0 to 50. f) Oil fate plot for days 0 to 20 after the oil spill. Yellow-displaced oil; Green-evaporated oil; Red-oil trapped on coast; Blue-oil at the surface. g) Plot showing the length of affected coast 0 to 20 days after the initial oil spill according to the MEDSLIK results. Figure was generated by the authors using MEDSLIK (http://oceanography.ucy.ac.cy/medslik/).

Multidisciplinary oil spill modeling to protect coastal communities and the environment of the Eastern Mediterranean Sea

Tiago M. Alves[1,*], Eleni Kokinou[2], George Zodiatis[3], Hari Radhakrishnan[3], Costas Panagiotakis[4] and Robin Lardner[3]

1) 3D Seismic Lab - School of Earth and Ocean Sciences, Cardiff University - Main Building, Park Place, Cardiff, CF10 3AT, UK (alvest@cardiff.ac.uk)
2) Department of Environmental and Natural Resources Engineering, Technological Educational Institute Crete, 3 Romanou Str. Chalepa, Chania, Crete GR 73133, Greece (ekokinou@staff.teicrete.gr)
3) Oceanography Centre, University of Cyprus, P.O. Box 20537, 1678 Nicosia, Cyprus (gzodiac@ucy.ac.cy)
4) Department of Business Administration, Technological Educational Institute Crete, Agios Nikolaos, Greece (cpanag@staff.teicrete.gr)

Oil Fate for Worst-Case Scenario: Site 13

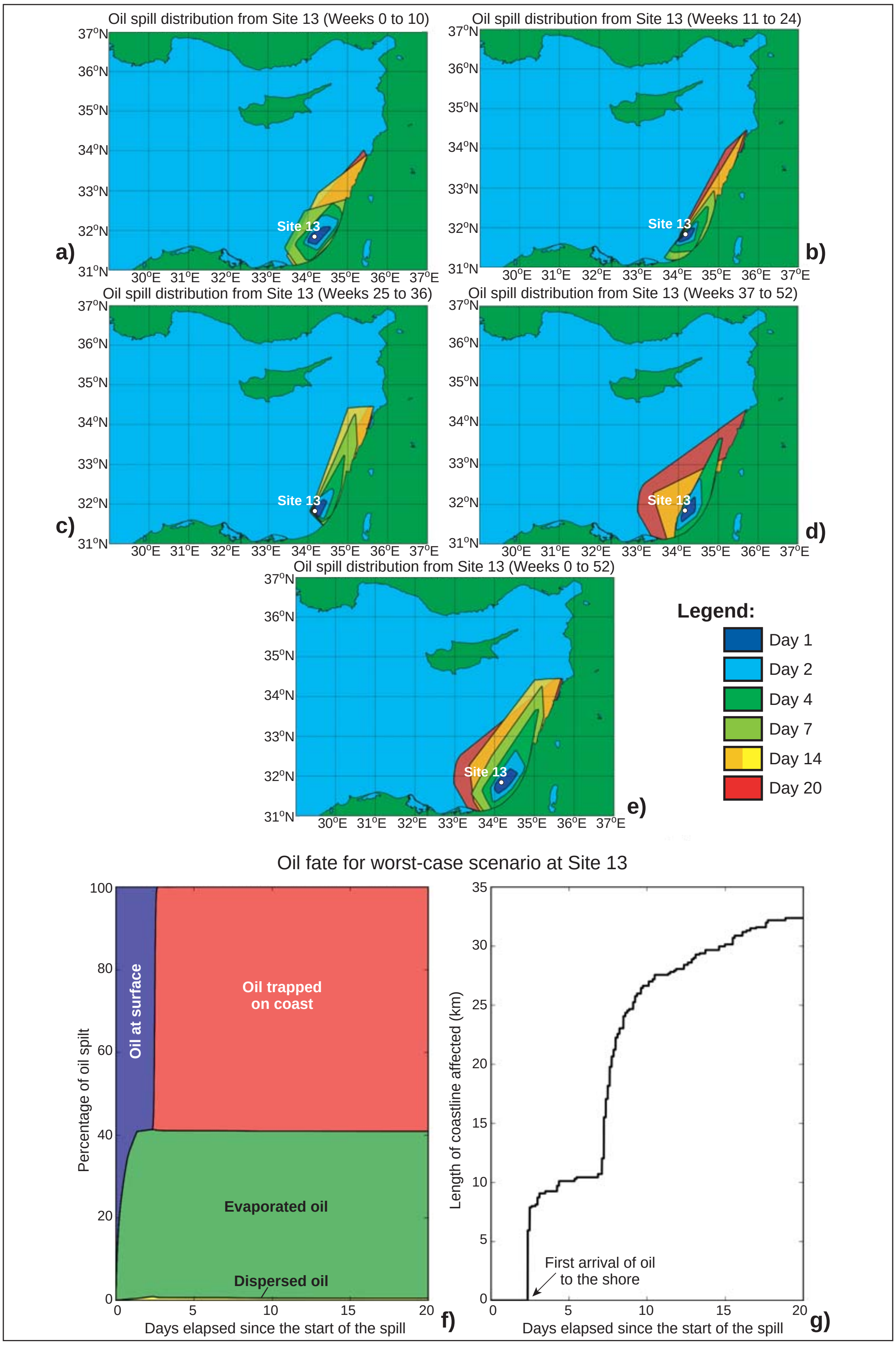

Supplementary Figure 13 - MEDSLIK model results for Site 13 at the Mari-B oil and gas platform offshore Israel. a) Oil spill distribution in weeks 0 to 10. b) Oil spill distribution in weeks 12 to 24. c) Oil spill distribution in weeks 25 to 36. d) Oil spill distribution in weeks 37 to 52. e) Oil spill distribution considering average meteorological and oceanographic conditions for weeks 0 to 50. f) Oil fate plot for days 0 to 20 after the oil spill. Yellow-displaced oil; Green-evaporated oil; Red-oil trapped on coast; Blue-oil at the surface. g) Plot showing the length of affected coast 0 to 20 days after the initial oil spill according to the MEDSLIK results. Figure was generated by the authors using MEDSLIK (http://oceanography.ucy.ac.cy/medslik/).

Multidisciplinary oil spill modeling to protect coastal communities and the environment of the Eastern Mediterranean Sea

Tiago M. Alves[1,*], Eleni Kokinou[2], George Zodiatis[3], Hari Radhakrishnan[3], Costas Panagiotakis[4] and Robin Lardner[3]

1) 3D Seismic Lab - School of Earth and Ocean Sciences, Cardiff University - Main Building, Park Place, Cardiff, CF10 3AT, UK (alvest@cardiff.ac.uk)
2) Department of Environmental and Natural Resources Engineering, Technological Educational Institute Crete, 3 Romanou Str. Chalepa, Chania, Crete GR 73133, Greece (ekokinou@staff.teicrete.gr)
3) Oceanography Centre, University of Cyprus, P.O. Box 20537, 1678 Nicosia, Cyprus (gzodiac@ucy.ac.cy)
4) Department of Business Administration, Technological Educational Institute Crete, Agios Nikolaos, Greece (cpanag@staff.teicrete.gr)

Oil Fate for Worst-Case Scenario: Site 14

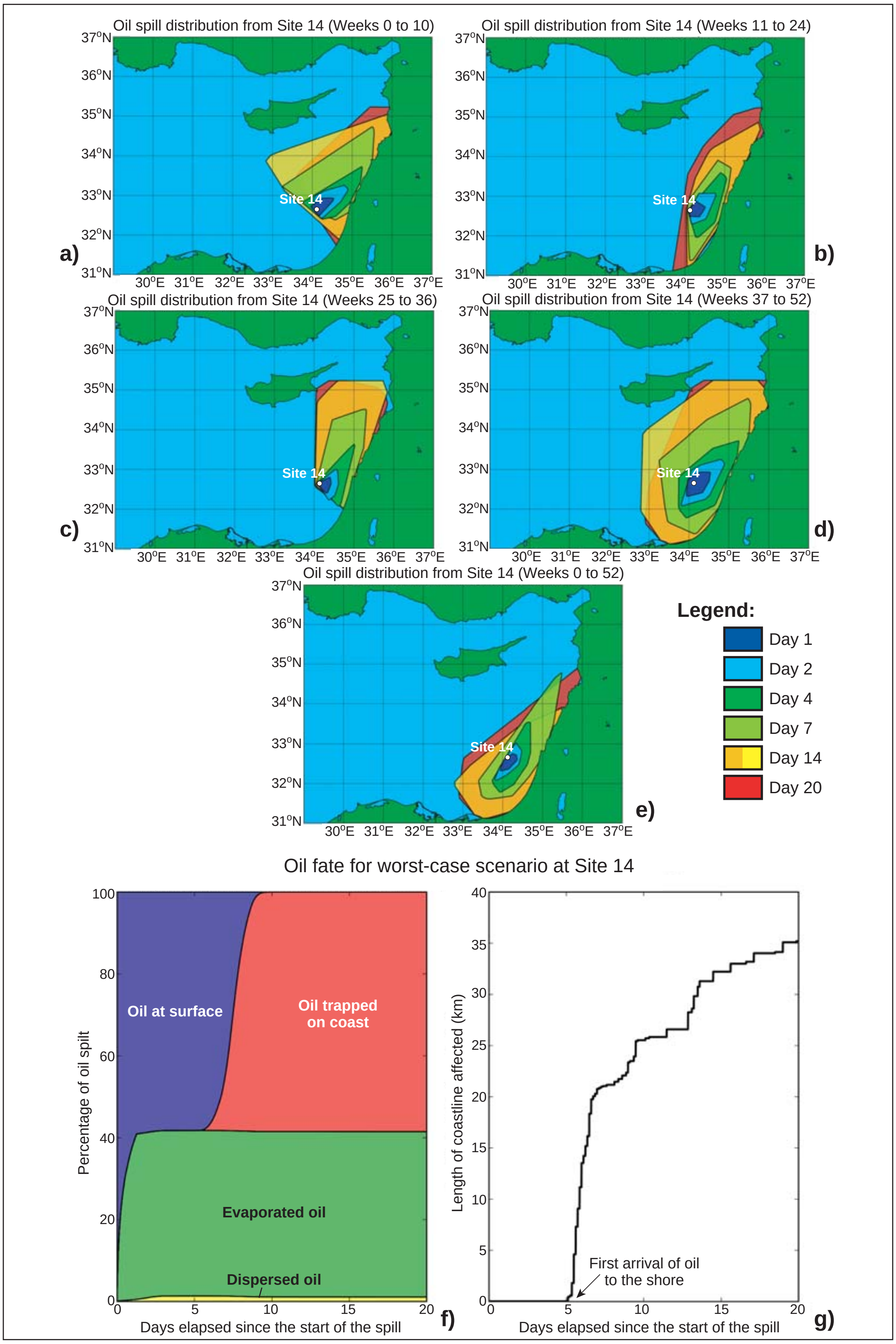

Supplementary Figure 14 - MEDSLIK model results for Site 14, which coincides with the location of the Dalit oil and gas platform, offshore Israel. a) Oil spill distribution in weeks 0 to 10. b) Oil spill distribution in weeks 12 to 24. c) Oil spill distribution in weeks 25 to 36. d) Oil spill distribution in weeks 37 to 52. e) Oil spill distribution considering average meteorological and oceanographic conditions for weeks 0 to 50. f) Oil fate plot for days 0 to 20 after the oil spill. Yellow-displaced oil; Green-evaporated oil; Red-oil trapped on coast; Blue-oil at the surface. g) Plot showing the length of affected coast 0 to 20 days after the initial oil spill according to the MEDSLIK results. Figure was generated by the authors using MEDSLIK (http://oceanography.ucy.ac.cy/medslik/).

Multidisciplinary oil spill modeling to protect coastal communities and the environment of the Eastern Mediterranean Sea

Tiago M. Alves[1,*], Eleni Kokinou[2], George Zodiatis[3], Hari Radhakrishnan[3], Costas Panagiotakis[4] and Robin Lardner[3]

1) 3D Seismic Lab - School of Earth and Ocean Sciences, Cardiff University - Main Building, Park Place, Cardiff, CF10 3AT, UK (alvest@cardiff.ac.uk)
2) Department of Environmental and Natural Resources Engineering, Technological Educational Institute Crete, 3 Romanou Str. Chalepa, Chania, Crete GR 73133, Greece (ekokinou@staff.teicrete.gr)
3) Oceanography Centre, University of Cyprus, P.O. Box 20537, 1678 Nicosia, Cyprus (gzodiac@ucy.ac.cy)
4) Department of Business Administration, Technological Educational Institute Crete, Agios Nikolaos, Greece (cpanag@staff.teicrete.gr)

Oil Fate for Worst-Case Scenario: Site 15

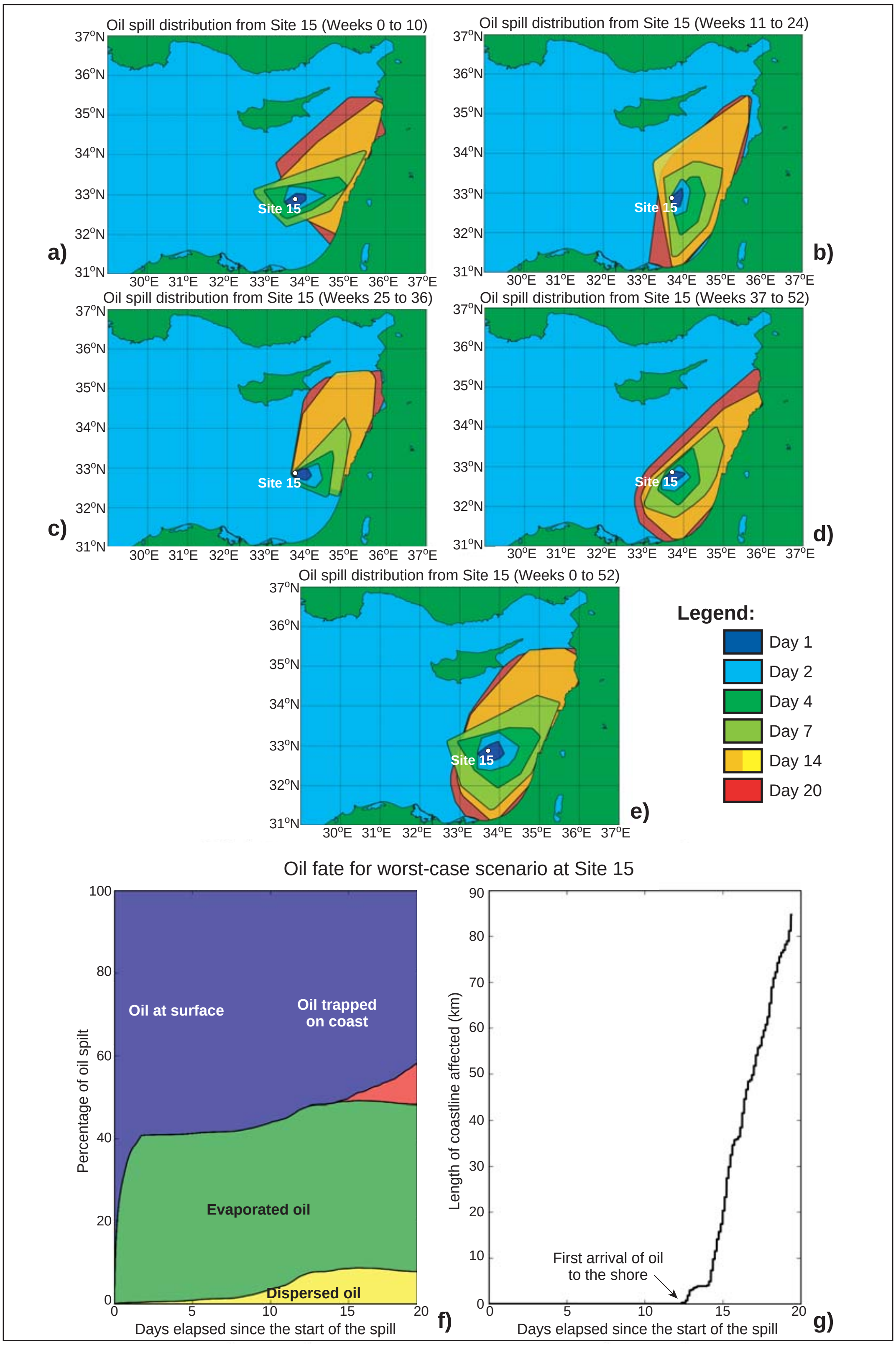

Supplementary Figure 15 - MEDSLIK model results for Site 15, located at the Tamar platform, offshore Israel. a) Oil spill distribution in weeks 0 to 10. b) Oil spill distribution in weeks 12 to 24. c) Oil spill distribution in weeks 25 to 36. d) Oil spill distribution in weeks 37 to 52. e) Oil spill distribution considering average meteorological and oceanographic conditions for weeks 0 to 50. f) Oil fate plot for days 0 to 20 after the oil spill. Yellow-displaced oil; Green-evaporated oil; Red-oil trapped on coast; Blue-oil at the surface. g) Plot showing the length of affected coast 0 to 20 days after the initial oil spill according to the MEDSLIK results. Figure was generated by the authors using MEDSLIK (http://oceanography.ucy.ac.cy/medslik/).

Multidisciplinary oil spill modeling to protect coastal communities and the environment of the Eastern Mediterranean Sea

Tiago M. Alves[1,*], Eleni Kokinou[2], George Zodiatis[3], Hari Radhakrishnan[3], Costas Panagiotakis[4] and Robin Lardner[3]

1) 3D Seismic Lab - School of Earth and Ocean Sciences, Cardiff University - Main Building, Park Place, Cardiff, CF10 3AT, UK (alvest@cardiff.ac.uk)
2) Department of Environmental and Natural Resources Engineering, Technological Educational Institute Crete, 3 Romanou Str. Chalepa, Chania, Crete GR 73133, Greece (ekokinou@staff.teicrete.gr)
3) Oceanography Centre, University of Cyprus, P.O. Box 20537, 1678 Nicosia, Cyprus (gzodiac@ucy.ac.cy)
4) Department of Business Administration, Technological Educational Institute Crete, Agios Nikolaos, Greece (cpanag@staff.teicrete.gr)

Oil Fate for Worst-Case Scenario: Site 16

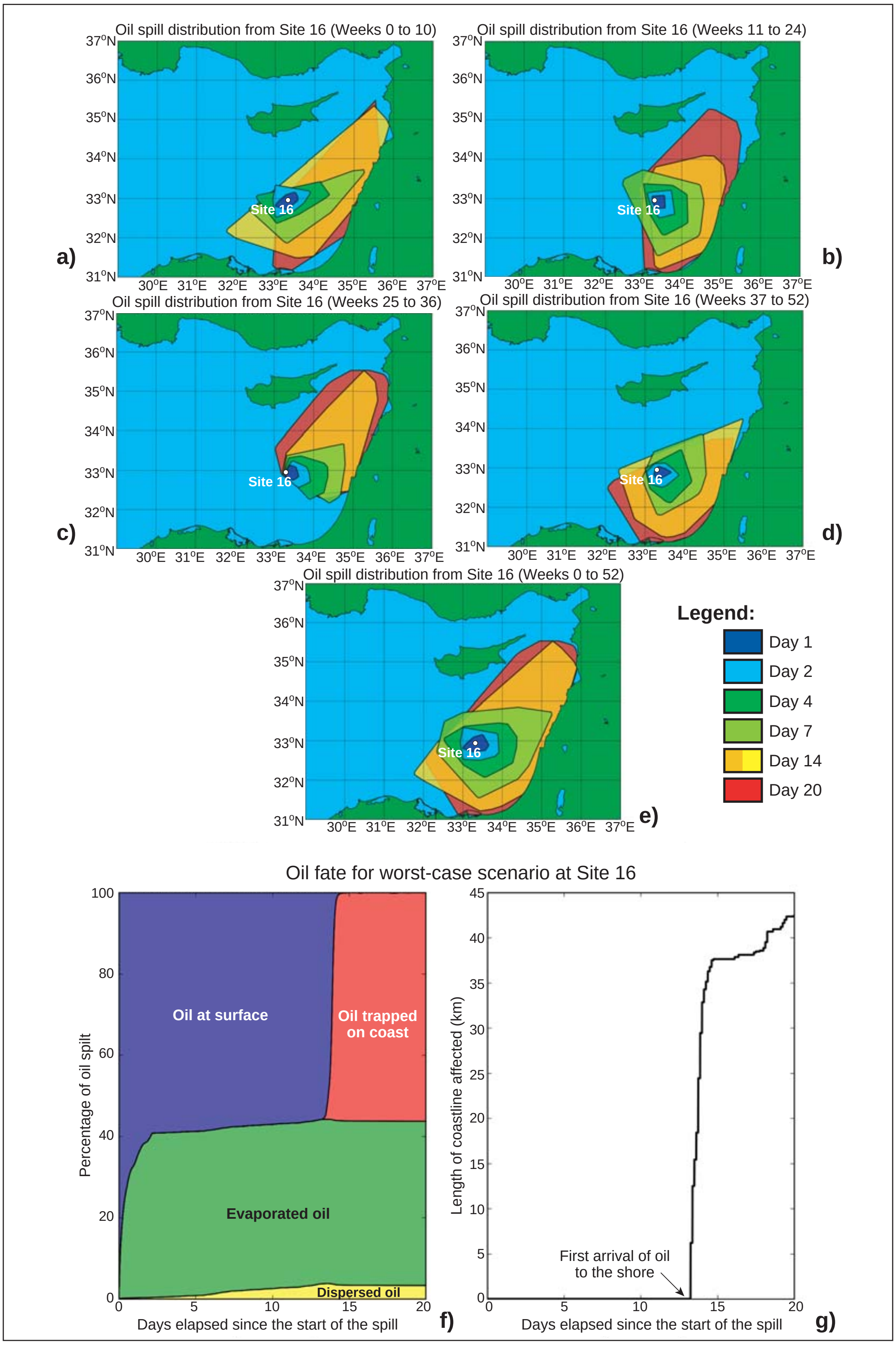

Supplementary Figure 16 - MEDSLIK model results for Site 16, located at the Leviathan platform, offshore Israel. a) Oil spill distribution in weeks 0 to 10. b) Oil spill distribution in weeks 12 to 24. c) Oil spill distribution in weeks 25 to 36. d) Oil spill distribution in weeks 37 to 52. e) Oil spill distribution considering average meteorological and oceanographic conditions for weeks 0 to 50. f) Oil fate plot for days 0 to 20 after the oil spill. Yellow-displaced oil; Green-evaporated oil; Red-oil trapped on coast; Blue-oil at the surface. g) Plot showing the length of affected coast 0 to 20 days after the initial oil spill according to the MEDSLIK results. Figure was generated by the authors using MEDSLIK (http://oceanography.ucy.ac.cy/medslik/).

Multidisciplinary oil spill modeling to protect coastal communities and the environment of the Eastern Mediterranean Sea

Tiago M. Alves[1,*], Eleni Kokinou[2], George Zodiatis[3], Hari Radhakrishnan[3], Costas Panagiotakis[4] and Robin Lardner[3]

1) 3D Seismic Lab - School of Earth and Ocean Sciences, Cardiff University - Main Building, Park Place, Cardiff, CF10 3AT, UK (alvest@cardiff.ac.uk)
2) Department of Environmental and Natural Resources Engineering, Technological Educational Institute Crete, 3 Romanou Str. Chalepa, Chania, Crete GR 73133, Greece (ekokinou@staff.teicrete.gr)
3) Oceanography Centre, University of Cyprus, P.O. Box 20537, 1678 Nicosia, Cyprus (gzodiac@ucy.ac.cy)
4) Department of Business Administration, Technological Educational Institute Crete, Agios Nikolaos, Greece (cpanag@staff.teicrete.gr)

Oil Fate for Worst-Case Scenario: Site 17

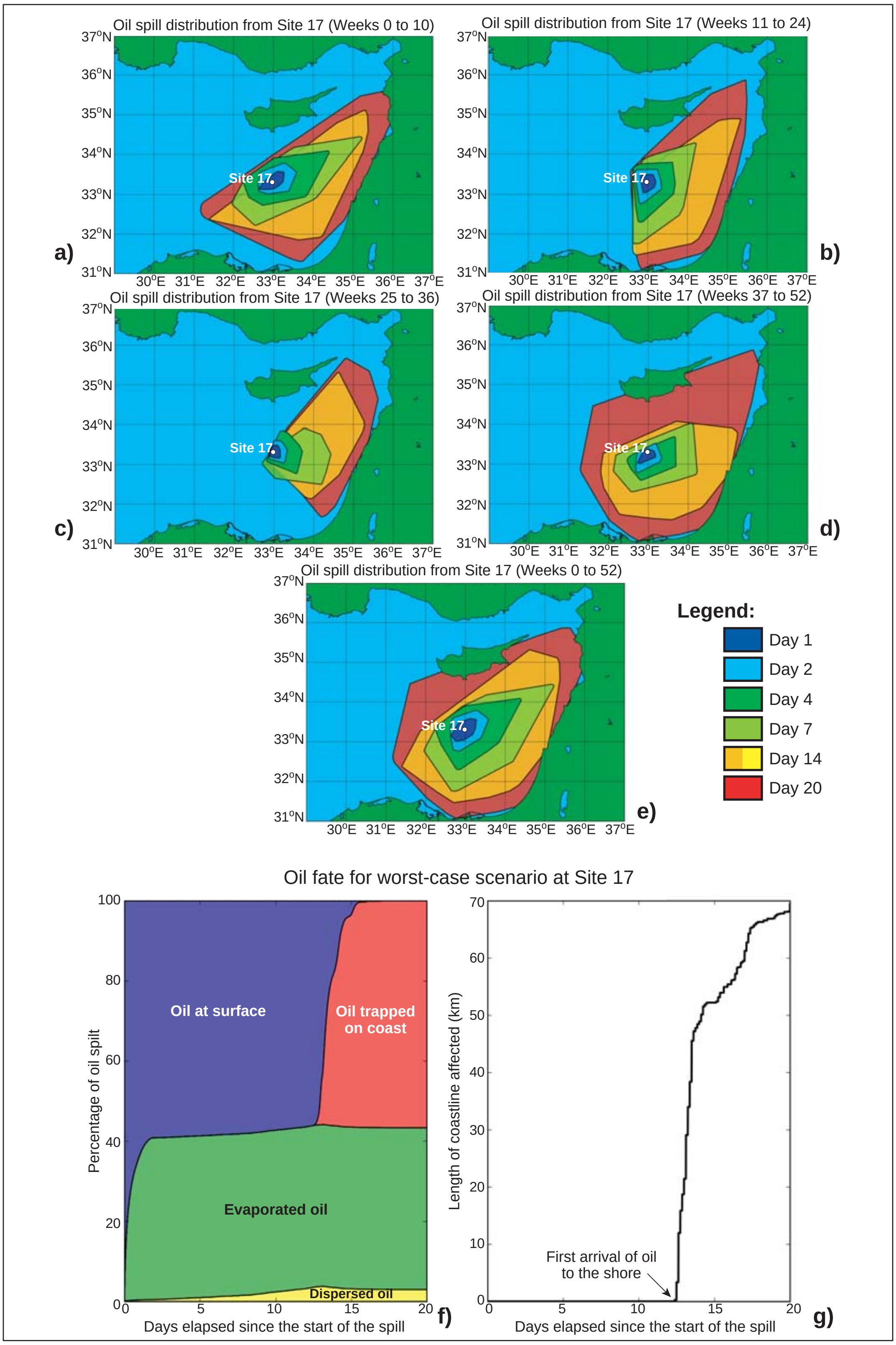

Supplementary Figure 17 - MEDSLIK model results for Site 17 at the Aphrodite Field, offshore Cyprus. a) Oil spill distribution in weeks 0 to 10. b) Oil spill distribution in weeks 12 to 24. c) Oil spill distribution in weeks 25 to 36. d) Oil spill distribution in weeks 37 to 52. e) Oil spill distribution considering average meteorological and oceanographic conditions for weeks 0 to 50. f) Oil fate plot for days 0 to 20 after the oil spill. Yellow-displaced oil; Green-evaporated oil; Red-oil trapped on coast; Blue-oil at the surface. g) Plot showing the length of affected coast 0 to 20 days after the initial oil spill according to the MEDSLIK results. Figure was generated by the authors using MEDSLIK (http://oceanography.ucy.ac.cy/medslik/).

Multidisciplinary oil spill modeling to protect coastal communities and the environment of the Eastern Mediterranean Sea

Tiago M. Alves[1,*], Eleni Kokinou[2], George Zodiatis[3], Hari Radhakrishnan[3], Costas Panagiotakis[4] and Robin Lardner[3]

1) 3D Seismic Lab - School of Earth and Ocean Sciences, Cardiff University - Main Building, Park Place, Cardiff, CF10 3AT, UK (alvest@cardiff.ac.uk)
2) Department of Environmental and Natural Resources Engineering, Technological Educational Institute Crete, 3 Romanou Str. Chalepa, Chania, Crete GR 73133, Greece (ekokinou@staff.teicrete.gr)
3) Oceanography Centre, University of Cyprus, P.O. Box 20537, 1678 Nicosia, Cyprus (gzodiac@ucy.ac.cy)
4) Department of Business Administration, Technological Educational Institute Crete, Agios Nikolaos, Greece (cpanag@staff.teicrete.gr)

Oil Fate for Worst-Case Scenario: Site 18

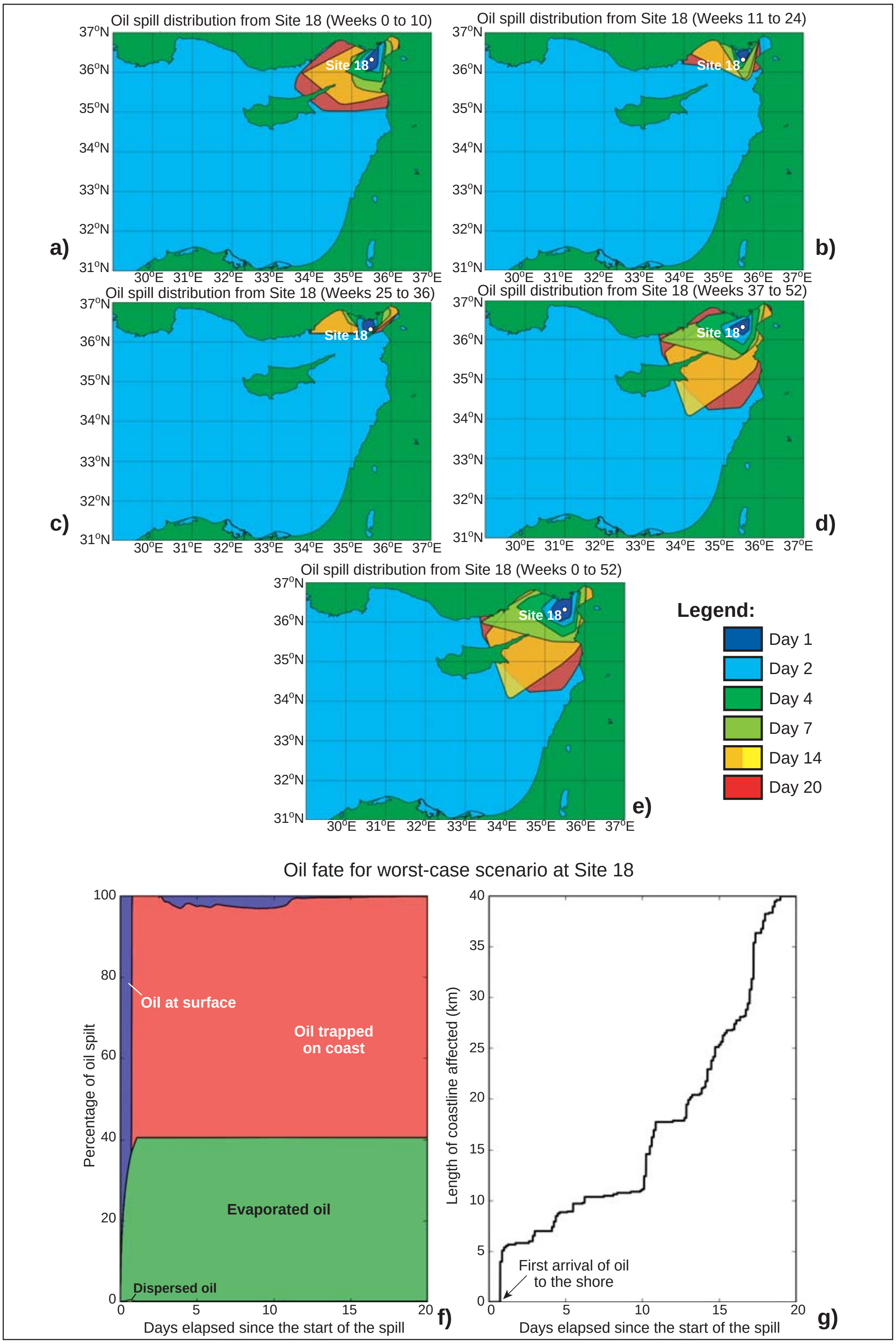

Supplementary Figure 18 - MEDSLIK model results for Site 18 representing a shipping accident 25 nautical miles from the Ceyhan Port, SE Turkey. a) Oil spill distribution in weeks 0 to 10. b) Oil spill distribution in weeks 12 to 24. c) Oil spill distribution in weeks 25 to 36. d) Oil spill distribution in weeks 37 to 52. e) Oil spill distribution considering average meteorological and oceanographic conditions for weeks 0 to 50. f) Oil fate plot for days 0 to 20 after the oil spill. Yellow-displaced oil; Green-evaporated oil; Red-oil trapped on coast; Blue-oil at the surface. g) Plot showing the length of affected coast 0 to 20 days after the initial oil spill according to the MEDSLIK results. Figure was generated by the authors using MEDSLIK (http://oceanography.ucy.ac.cy/medslik/).

Multidisciplinary oil spill modeling to protect coastal communities and the environment of the Eastern Mediterranean Sea

Tiago M. Alves[1,*], Eleni Kokinou[2], George Zodiatis[3], Hari Radhakrishnan[3], Costas Panagiotakis[4] and Robin Lardner[3]

1) 3D Seismic Lab - School of Earth and Ocean Sciences, Cardiff University - Main Building, Park Place, Cardiff, CF10 3AT, UK (alvest@cardiff.ac.uk)
2) Department of Environmental and Natural Resources Engineering, Technological Educational Institute Crete, 3 Romanou Str. Chalepa, Chania, Crete GR 73133, Greece (ekokinou@staff.teicrete.gr)
3) Oceanography Centre, University of Cyprus, P.O. Box 20537, 1678 Nicosia, Cyprus (gzodiac@ucy.ac.cy)
4) Department of Business Administration, Technological Educational Institute Crete, Agios Nikolaos, Greece (cpanag@staff.teicrete.gr)

Oil Fate for Worst-Case Scenario: Site 19

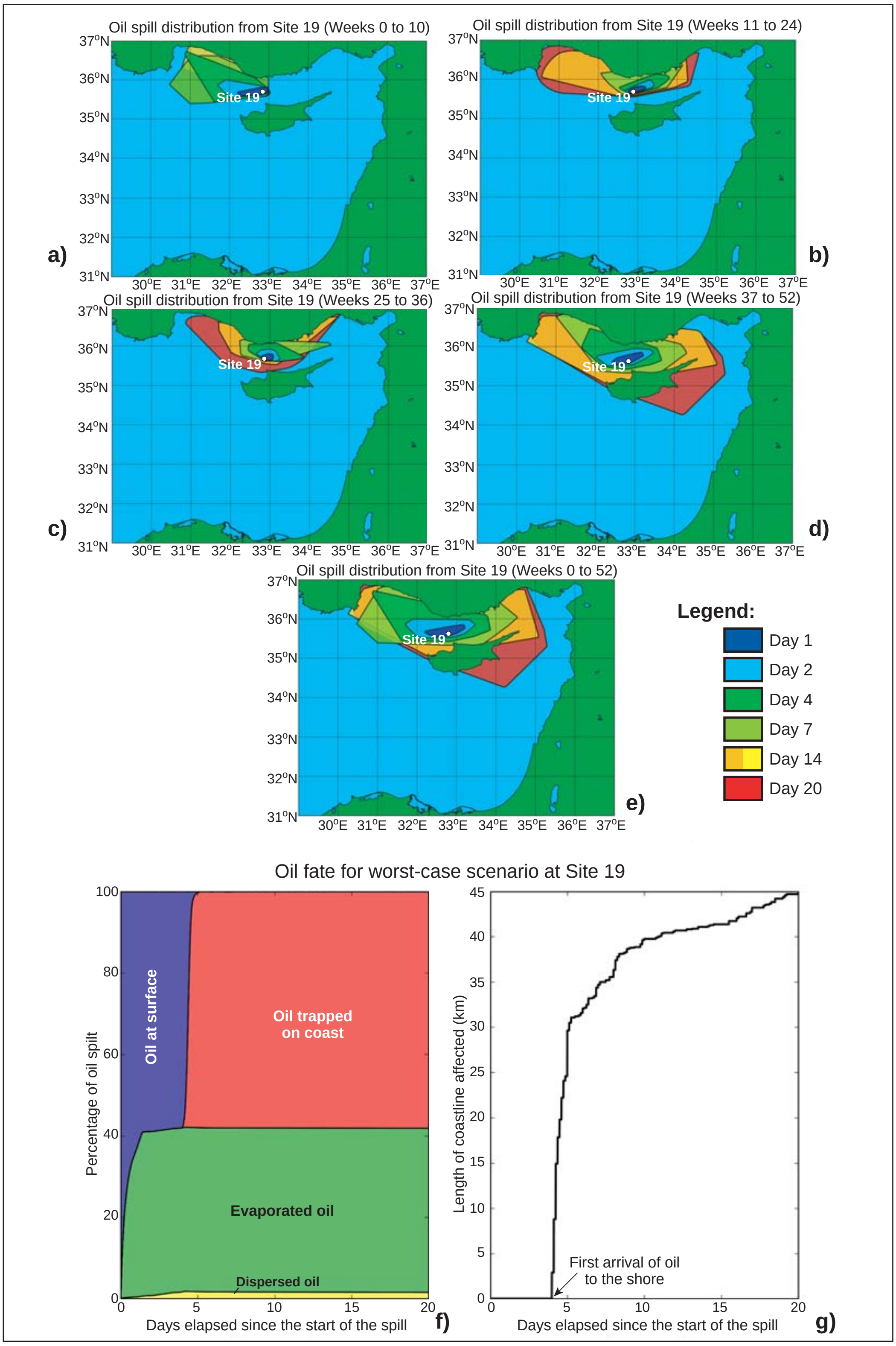

Supplementary Figure 19 - MEDSLIK model results for Site 19 representing a shipping accident 10 nautical miles from the Anamur Port, between Turkey and Cyprus. a) Oil spill distribution in weeks 0 to 10. b) Oil spill distribution in weeks 12 to 24. c) Oil spill distribution in weeks 25 to 36. d) Oil spill distribution in weeks 37 to 52. e) Oil spill distribution considering average meteorological and oceanographic conditions for weeks 0 to 50. f) Oil fate plot for days 0 to 20 after the oil spill. Yellow-displaced oil; Green-evaporated oil; Red-oil trapped on coast; Blue-oil at the surface. g) Plot showing the length of affected coast 0 to 20 days after the initial oil spill according to the MEDSLIK results. Figure was generated by the authors using MEDSLIK (http://oceanography.ucy.ac.cy/medslik/).
