# Supplementary material for: Multidisciplinary oil spill modeling to protect coastal communities and the environment of the Eastern Mediterranean Sea
Source: Sci Rep. 2016 Nov 10;6:36882. doi: 10.1038/srep36882 (PMC5103274; doi:10.1038/srep36882)
Supplement: Supplementary Information [file srep36882-s2.doc]

**Multidisciplinary oil spill modeling to protect coastal communities and the environment of the Eastern Mediterranean Sea**

Tiago M. Alves^1,*^, Eleni Kokinou^2^, George Zodiatis^3^, Hari Radhakrishnan^3^,

Costas Panagiotakis^4^ and Robin Lardner^3^

1) 3D Seismic Lab – School of Earth and Ocean Sciences, Cardiff University – Main Building, Park Place, Cardiff, CF10 3AT, United Kingdom ([alvest@cardiff.ac.uk](mailto:alvest@cardiff.ac.uk))

2) Department of Environmental and Natural Resources Engineering, Technological Educational Institute Crete, 3 Romanou Str. Chalepa, Chania, Crete GR 73133, Greece (ekokinou@staff.teicrete.gr)

3) Oceanography Centre, University of Cyprus, P.O. Box 20537, 1678 Nicosia, Cyprus (gzodiac@ucy.ac.cy)

4) Department of Business Administration, Technological Educational Institute Crete, Agios Nikolaos, Greece (cpanag@staff.teicrete.gr)

Supplementary Table 1 – Location of 73 historic oil spills East of 29^o^E taken from the MEDESS 4MS access system at http://www.medess4ms.eu/ and ([http://medgismar.rempec.org/#).](http://medgismar.rempec.org/%23).)

| **Number** | **Nearest Country** | **Relative Location** | **Local ESI Classification** | **Latitude (North)** | **Longitude (East)** | **Date** |
| --- | --- | --- | --- | --- | --- | --- |
| 1 | Cyprus | South of Akrotiri | ESI 9 | 34.72 | 33.32 | 28-Nov-77 |
| 2 | Cyprus | Limassol | ESI 8 | 34.92 | 33.65 | 07-Jun-80 |
| 3 | Syria | Latakia | ESI 8 | 35.53 | 35.72 | 27-Dec-81 |
| 4 | Lebanon | Tripoli | ESI 5B and ESI 6A | 34.45 | 35.77 | 02-Jan-82 |
| 5 | Egypt | Alexandria | ESI 9 | 31.15 | 29.88 | 01-Sep-82 |
| 6 | Israel | Tel Aviv | ESI 9 | 31.8 | 34.63 | 05-Jan-83 |
| 7 | Lebanon | West of Tyre | ESI 5B | 34 | 34.67 | 14-Feb-83 |
| 8 | State of Palestine | Gaza City | ESI 4 | 31.67 | 34.58 | 08-Jun-83 |
| 9 | Syria | South of Baniyas | ESI 3 | 34.45 | 35.83 | 03-Nov-83 |
| 10 | Syria | Baniyas | ESI 5B and ESI 6A | 35.25 | 35.95 | 29-Jun-85 |
| 11 | Egypt | El-Agamy | ESI 9 | 31.1 | 29.62 | 31-May-87 |
| 12 | Egypt | Gamasa | ESI 9 | 31.28 | 32.37 | 08-Aug-87 |
| 13 | Turkey | Mersin | ESI 9 | 36.78 | 34.62 | 01-Nov-87 |
| 14 | Cyprus | Southeast of Akrotiri | ESI 8 | 34.72 | 33.3 | 02-Oct-88 |
| 15 | Israel | Ashdod | ESI 4 | 31.8 | 34.65 | 19-Nov-88 |
| 16 | Cyprus | Girne | ESI 8 | 32.82 | 35 | 26-Feb-89 |
| 17 | Egypt | Sidi Kirayr | ESI 9 | 31.1 | 29.62 | 30-Mar-89 |
| 18 | Lebanon | Beirut | ESI 9 | 33.92 | 35.52 | 13-Apr-89 |
| 19 | Cyprus | South of Larnaca | ESI 8 | 34.67 | 33.03 | 28-Apr-89 |
| 20 | Lebanon | North of Tyre | ESI 5B | 34 | 35.58 | 15-Jun-89 |
| 21 | Lebanon | South Beirut | ESI 5B | 34.05 | 35.37 | 09-Jul-89 |
| 22 | Lebanon | Beirut | ESI 9 | 34.03 | 35.47 | 23-Jul-89 |
| 23 | Lebanon | Beirut | ESI 5B | 34.07 | 35.4 | 29-Aug-89 |
| 24 | Israel | Tel Aviv | ESI 9 | 32.2 | 34.78 | 30-Nov-89 |
| 25 | Cyprus | Vasilikos Depot | ESI 8 | 34.72 | 33.3 | 29-Apr-90 |
| 26 | Lebanon | Beirut | ESI 5B and ESI 6A | 33.98 | 35.63 | 21-Sep-90 |
| 27 | Israel | North of Haifa | ESI 8 | 32.83 | 35.03 | 14-Aug-91 |
| 28 | Lebanon | Tripoli | ESI 5B and ESI 6A | 34.47 | 35.83 | 18-Aug-91 |
| 29 | Israel | North of Haifa | ESI 8 | 32.82 | 35 | 27-Jan-92 |
| 30 | Lebanon | North of Tyre | ESI 5B | 33.67 | 35.4 | 14-Apr-92 |
| 31 | Egypt | El-Alamein | ESI 3 | 31.1 | 29.62 | 14-Apr-92 |
| 32 | Lebanon | Tripoli | ESI 5B and ESI 6A | 34.47 | 35.83 | 01-Jun-92 |
| 33 | Egypt | Gamasa | ESI 9 | 31.43 | 32.37 | 27-Oct-92 |
| 34 | Egypt | Alexandria | ESI 9 | 31.13 | 29.6 | 09-Nov-92 |
| 35 | Egypt | Alexandria | ESI 9 | 31.15 | 29.6 | 21-Feb-93 |
| 36 | Turkey | Gulf of Antalya | ESI 9 | 36.82 | 30.62 | 25-Mar-93 |
| 37 | Lebanon | North of Tyre | ESI 5B | 33.9 | 35.52 | 27-Apr-93 |
| 38 | Lebanon | North of Tyre | ESI 5B | 33.9 | 35.52 | 03-May-93 |
| 39 | SE Greece | East of Kastelorizo isl. | ESI 9 | 36.5 | 29.1 | 09-Jul-93 |
| 40 | Lebanon | Tripoli | ESI 5B and ESI 6A | 34.42 | 35.82 | 06-Mar-94 |
| 41 | Turkey | SE of Finike | ESI 9 | 36.38 | 30.67 | 01-May-94 |
| 42 | Egypt | El-Agamy | ESI 9 | 31.15 | 29.88 | 02-Jul-94 |
| 43 | Israel | West of Haifa | ESI 8 | 33.33 | 33.67 | 09-Aug-94 |
| 44 | Greece | South of Kastelorizo isl. | ESI 9 | 35.55 | 29.35 | 21-Nov-94 |
| 45 | Israel | Ashdod | ESI 4 | 31.82 | 34.65 | 25-May-95 |
| 46 | Israel | West of Tel Aviv | ESI 3 | 32.83 | 34.14 | 02-Jun-95 |
| 47 | Turkey | South of Antalya | ESI 9 | 36.08 | 31.05 | 25-Aug-95 |
| 48 | Israel | Haifa | ESI 8 | 32.82 | 35 | 23-Oct-95 |
| 49 | Egypt | Alexandria | ESI 9 | 31.15 | 29.88 | 30-Oct-95 |
| 50 | Cyprus | Ammochostos Bay | ESI 8 | 35.12 | 33.95 | 31-Jan-96 |
| 51 | Egypt | Gamasa | ESI 9 | 31.28 | 32.3 | 18-Oct-97 |
| 52 | Israel | Tel Aviv | ESI 9 | 31.82 | 34.63 | 12-Feb-98 |
| 53 | Syria | Latakia | ESI 8 | 35.53 | 35.92 | 16-Mar-98 |
| 54 | Turkey | Samandag, SE Turkey | ESI 3 | 35.92 | 35.5 | 18-Mar-98 |
| 55 | Lebanon | Beirut | ESI 5B | 33.92 | 35.52 | 01-Nov-98 |
| 56 | Israel | Haifa | ESI 8 | 32.87 | 35 | 16-Dec-98 |
| 57 | State of Palestine | Gaza City | ESI 4 | 31.67 | 34.53 | 04-Jan-99 |
| 58 | Cyprus | South of Larnaca | ESI 8 | 34.7 | 34 | 28-Jul-99 |
| 59 | Cyprus | South of Larnaca | ESI 8 | 34.4 | 34.1 | 31-Dec-99 |
| 60 | Lebanon | South of Tyre | ESI 3 | 32.82 | 35 | 10-Apr-00 |
| 61 | Egypt | Al-Alamein | ESI 9 | 31.27 | 30.1 | 06-May-00 |
| 62 | Cyprus | Ammochostos Bay | ESI 8 | 34.05 | 35.22 | 11-Dec-00 |
| 63 | Turkey | Gulf of Antalya | ESI 9 | 36.73 | 30.76 | 31-Dec-00 |
| 64 | Turkey | Mersin | ESI 9 | 36.8 | 34.64 | 20-Jan-12 |
| 65 | Syria | Latakia | ESI 8 | 35.57 | 35.73 | 28-Feb-12 |
| 66 | Turkey | Mersin | ESI 9 | 36.8 | 34.64 | 31-Mar-12 |
| 67 | Greece | South of Kastelorizo isl. | ESI 9 | 36.15 | 29.55 | 21-Oct-12 |
| 68 | Turkey | Aydincik, Mersin | ESI 3 and ESI 4 | 36.15 | 33.53 | 09-Jan-13 |
| 69 | Turkey | Aydincik, Mersin | ESI 3 and ESI 4 | 36.18 | 33.52 | 04-Mar-13 |
| 70 | Cyprus | Girne | ESI 8 | 35.58 | 33.45 | 12-Mar-13 |
| 71 | Egypt | Alexandria | ESI 9 | 31.19 | 29.86 | 13-Sep-13 |
| 72 | Lebanon | Jyeh Power Station | ESI 5B and ESI 6A | 33.6667 | 35.4125 | 13-Jul-06 |
| 73 | Cyprus | Ammochostos Bay | ESI 8 | 35.31066 | 33.9916 | 15-Jul-13 |
