# Supplementary material for: Multidisciplinary oil spill modeling to protect coastal communities and the environment of the Eastern Mediterranean Sea
Source: Sci Rep. 2016 Nov 10;6:36882. doi: 10.1038/srep36882 (PMC5103274; doi:10.1038/srep36882)
Supplement: Supplementary Information [file srep36882-s3.doc]

**Multidisciplinary oil spill modeling to protect coastal communities and the environment of the Eastern Mediterranean Sea**

Tiago M. Alves^1,*^, Eleni Kokinou^2^, George Zodiatis^3^, Hari Radhakrishnan^3^,

Costas Panagiotakis^4^ and Robin Lardner^3^

1) 3D Seismic Lab – School of Earth and Ocean Sciences, Cardiff University – Main Building, Park Place, Cardiff, CF10 3AT, United Kingdom ([alvest@cardiff.ac.uk](mailto:alvest@cardiff.ac.uk))

2) Department of Environmental and Natural Resources Engineering, Technological Educational Institute Crete, 3 Romanou Str. Chalepa, Chania, Crete GR 73133, Greece (ekokinou@staff.teicrete.gr)

3) Oceanography Centre, University of Cyprus, P.O. Box 20537, 1678 Nicosia, Cyprus (gzodiac@ucy.ac.cy)

4) Department of Business Administration, Technological Educational Institute Crete, Agios Nikolaos, Greece (cpanag@staff.teicrete.gr)

Supplementary Table 2 - Integrated interpretation of the oil spill modelling based on the data in Figures 2, 3 and 4 and in Supplementary Figures 1 to 19.

| Site of  oil spill simulations | Areas according to Figure 2 where the oil spill is expanded | Orientation  of the spill | Prevailing bathymetric Slopes (^o^) | Prevailing bathymetric Aspects (^o^) | Prevailing direction (with respect to the North) of bathymetric features | Length (km) of  affected coast after 20 days | Comments on oil fate for a period of up to 20 days |
| --- | --- | --- | --- | --- | --- | --- | --- |
| 1 | 2, 3, 4 | Major axis: E-W  Minor axis: N-S | 0-20 and 40-60 | 0-25 (N-NNE) and  345-359 (NNW-N) | E-W | 22.5 | In the first 2 days the oil slick remains at surface and is evaporated. Very low (up to 1%) percent of dispersion. From 3rd to 20th day: oil slick at the surface, up to the 4th day. Continuous evaporation and expansion to the coast. |
| 2 | 2, 3, 4 | Major axis: NE-SW  Minor axis: SW-NE | 0-20 and 40-60 | 0-25 (N-NNE),  345-359 (NNW-N)  and 180-250 (S-SW) | NE-SW | 68 | In the first 6 days oil slick remains at surface and is evaporated. Very low (up to 1-2%) percent of dispersion. From 7th to 20th day: oil slick at the surface until the day 7 1/2. Continuous evaporation, dispersion (<1%) and expansion towards the coast. |
| 3 | 2, 3, 4 | Major axis: E-W  Minor axis: N-S | 0-20 and 40-60 | 0-25 (N-NNE),  345-359 (NNW-N)  and 180-250 (S-SW) | E-W and NE-SW | 36 | In the first 5 days oil slick remains at surface and is evaporated. Very low (up to 1-2%) percent of dispersion. From 6th to 20th day: Continuous evaporation, dispersion (<1%) up to day 12 1/2 day and expansion towards the coast. |
| 4 | 2, 3, 4 | Major axis: E-W  Minor axis: N-S | 0-20 and 40-60 | 0-25 (N-NNE),  345-359 (NNW-N)  and 180-250 (S-SW) | E-W | 27.5 | In the first 2 days oil slick remains at surface and is evaporated. Very low (up to 1-2%) percent of dispersion. From 3th to 20th day: Continuous evaporation and expansion to the coast. |
| 5 | 1, 2, 3, 4 | Major axis: NE-SW  Minor axis: SW-NE | 0-20 and 40-60 | 0-25 (N-NNE)  275-300 (W-NW)  345-359 (NNW-N) | NE-SW | 58 | In the first 4 days oil slick remains at surface and is evaporated. Very low (up to 1-2%) percent of dispersion. From 5th to 20th day: Continuous evaporation and expansion towards the coast. |
| 6 | 1, 2, 3, 4 | Major axis: NE-SW  Minor axis: SW-NE | 0-20 and 40-60 | 0-25 (N-NNE)  275-300 (W-NW)  345-359 (NNW-N) | NE-SW | 37 | In the first 2 days oil slick remains at surface and is evaporated. Very low (up to 1-2%) percent of dispersion. From 3rd to 20th day: Continuous evaporation and expansion towards the coast. |
| 7 | 1, 2, 3, 4 | Major axis: NE-SW  Minor axis: SW-NE | 0-20 and 40-60 | 0-25 (N-NNE)  275-300 (W-NW)  345-359 (NNW-N) | NE-SW | 48 | In the first 2 days oil slick remains at surface and is evaporated. Very low (up to 1-2%) percent of dispersion. From 3rd to 20th day: Continuous evaporation and expansion towards the coast. |
| 8 | 1, 2, 3, 4 | Major axis: NE-SW  Minor axis: SW-NE | 0-20 and 40-60 | 0-25 (N-NNE)  275-300 (W-NW)  345-359 (NNW-N) | NE-SW | 96 | In the first 2 days oil slick remains at surface and is evaporated. Very low (up to 1%) percent of dispersion. From 3rd to 20th day: oil slick on surface up to the 8th day. Continuous evaporation and expansion towards the coast. |
| 9 | 1, 2, 3, 4 | Major axis: NE-SW  Minor axis: SW-NE | 0-20 and 40-60 | 0-25 (N-NNE)  275-300 (W-NW)  345-359 (NNW-N) | NE-SW | 116 | In the first 3.5 days oil slick remains at surface and is evaporated. Very low (up to 1-2%) percent of dispersion. From 4th to 20th day: Continuous evaporation and expansion towards the coast. |
| 10 | 1, 2, 3 | Major axis: NE-SW  Minor axis: SW-NE | 0-20 and 40-60 | 0-25 (N-NNE)  275-300 (W-NW)  345-359 (NNW-N) | NE-SW | 69 | In the first 1.5 days oil slick remains at surface and is evaporated. Very low (1-2%) percent of dispersion. From 2nd to 20th day: oil slick on surface up to 7.5th day. Continuous evaporation and expansion towards the coast. |
| 11 | 1, 2, 4, 5 | Major axis: NE-SW  Minor axis: SW-NE | 0-20, 40-60  and 60-80 | 0-25 (N-NNE)  125-175 (SE-S)  275-300 (W-NW)  345-359 (NNW-N) | NE-SW | 100 | In the first 12 days oil slick remains at surface and is evaporated. Low (up to 5%) percent of dispersion. From 13th to 20th day: Continuous evaporation and expansion towards the coast. The percent of dispersion increases (7%) up to day 12 1/2 day and then remains stable (5%) until day 20. |
| 12 | 1, 2 | Major axis: NE-SW  Minor axis: SW-NE | 0-20 and 40-60 | 0-25 (N-NNE)  345-359 (NNW-N)  275-300 (W-NW) | NE-SW | 21.5 | In the first 2.5 days oil slick remains at surface and is evaporated. Very low (up to 1-2%) percent of dispersion. From 3rd to 20th day: Continuous evaporation, dispersion (<1%) and expansion towards the coast. |
| 13 | 1, 2 | Major axis: NE-SW  Minor axis: SW-NE | 0-20 and 40-60 | 0-25 (N-NNE)  345-359 (NNW-N)  275-300 (W-NW) | NE-SW | 32.5 | In the first 2.5 days oil slick remains at surface and is evaporated. Very low (up to 1-2%) percent of dispersion. From 3rd to 20th day: Continuous evaporation, dispersion (<1%) and expansion towards the coast. |
| 14 | 1, 2, 5 | Major axis: NE-SW  Minor axis: SW-NE | 0-20, 40-60  and 60-80 | 0-25 (N-NNE)  125-175 (SE-S)  275-300 (W-NW)  345-359 (NNW-N) | NE-SW | 35 | In the first 8.5 days oil slick remains at surface and is evaporated. Very low (up to 1-2%) percent of dispersion. From 9th to 20th day: Continuous evaporation, dispersion (1%) and expansion towards the coast. |
| 15 | 1, 2, 5 | Major axis: NE-SW  Minor axis: SW-NE | 0-20, 40-60  and 60-80 | 0-25 (N-NNE)  125-175 (SE-S)  275-300 (W-NW)  345-359 (NNW-N) | NE-SW | 85 | Continuous evaporation and at surface up to day 20. Dispersion is very low (<1%) until day 5 and then increases (9-10%) up day 209. Expansion towards the coast starts the 15th day. |
| 16 | 1, 2, 4, 5 | Major axis: NE-SW  Minor axis: SW-NE | 0-20, 40-60  and 60-80 | 0-25 (N-NNE)  125-175 (SE-SSE)  275-300 (W-NW)  345-359 (NNW-N) | NE-SW | 42.5 | In the first 13 days oil slick remains at surface and is evaporated. Very low (up to 1%) percent of dispersion up to 5th day, then increasing (3-4%) up to day 20. From 14th to 20th day: Continuous evaporation and expansion towards the coast. |
| 17 | 1, 2, 3, 4, 5 | Major axis: NE-SW  Minor axis: SW-NE | 0-20, 40-60  and 60-80 | 0-25 (N-NNE)  125-175 (SE-S)  275-300 (W-NW)  345-359 (NNW-N) | NE-SW | 68 | In the first 12.5 days oil slick remains at surface and is evaporated. Very low (up to 1%) percent of dispersion up to 5th day, then increasing (3-4%) up to day 20. From 13th to 20th day: Continuous evaporation and expansion towards the coast. |
| 18 | 5, 6, 7 | Major axis: N-S  Minor axis: E-W | 0-20 and 40-80 | 125-175 (SE-S)  150-250(SE-SW)  300-359 (NW-N) | E-W and ENE-WSW | 40 | In the first 1.5 days oil slick remains at surface and is evaporated. Very low (up to 1%) percent of dispersion. From 2nd to 20th day: Continuous evaporation and expansion towards the coast. Oil slick remains at surface between 5th and 15th day. |
| 19 | 5, 6, 7 | Major axis: E-W  Minor axis: N-S | 0-20 and 40-80 | 125-175 (SE-S)  150-250(SE-SW)  300-359 (NW-N) | E-W and ENE-WSW | 45 | In the first 5 days oil slick remains at surface and is evaporated. From 6th to 20th day: Continuous evaporation and expansion towards the coast. Dispersion is approximately 2% in the 20 days. |
